# A novel approach to identify driver genes involved in androgen-independent prostate cancer

**DOI:** 10.1186/1476-4598-13-120

**Published:** 2014-05-23

**Authors:** Ellyn N Schinke, Victor Bii, Arun Nalla, Dustin T Rae, Laura Tedrick, Gary G Meadows, Grant D Trobridge

**Affiliations:** 1 Department of Pharmaceutical Sciences, Washington State University, Spokane, WA 99210-1495, USA; 2 School of Molecular Biosciences, Pullman, WA, USA

**Keywords:** Prostate cancer, Lentiviral vector, Mutagenesis screen, Shuttle vector, Androgen-independent prostate cancer, Proto-oncogene

## Abstract

**Background:**

Insertional mutagenesis screens have been used with great success to identify oncogenes and tumor suppressor genes. Typically, these screens use gammaretroviruses (γRV) or transposons as insertional mutagens. However, insertional mutations from replication-competent γRVs or transposons that occur later during oncogenesis can produce passenger mutations that do not drive cancer progression. Here, we utilized a replication-incompetent lentiviral vector (LV) to perform an insertional mutagenesis screen to identify genes in the progression to androgen-independent prostate cancer (AIPC).

**Methods:**

Prostate cancer cells were mutagenized with a LV to enrich for clones with a selective advantage in an androgen-deficient environment provided by a dysregulated gene(s) near the vector integration site. We performed our screen using an *in vitro* AIPC model and also an *in vivo* xenotransplant model for AIPC. Our approach identified proviral integration sites utilizing a shuttle vector that allows for rapid rescue of plasmids in *E. coli* that contain LV long terminal repeat (LTR)-chromosome junctions. This shuttle vector approach does not require PCR amplification and has several advantages over PCR-based techniques.

**Results:**

Proviral integrations were enriched near prostate cancer susceptibility loci in cells grown in androgen-deficient medium (p < 0.001), and five candidate genes that influence AIPC were identified; *ATPAF1*, *GCOM1*, *MEX3D*, *PTRF*, and *TRPM4*. Additionally, we showed that RNAi knockdown of *ATPAF1* significantly reduces growth (p < 0.05) in androgen-deficient conditions.

**Conclusions:**

Our approach has proven effective for use in PCa, identifying a known prostate cancer gene, PTRF, and also several genes not previously associated with prostate cancer. The replication-incompetent shuttle vector approach has broad potential applications for cancer gene discovery, and for interrogating diverse biological and disease processes.

## Background

Insertional mutagenesis screens using replicating retroviruses have identified many genes that contribute to cancer initiation and progression and have greatly improved our understanding of carcinogenesis (reviewed by Uren et al. [1]). These screens identify genomic loci which contain proviral integration sites that are identified from different tumors, called common insertion sites (CISs). These CISs occur because integrated retroviruses dysregulate nearby genes by a variety of mechanisms, and clones with provirus insertions near dysregulated genes that provide a selective advantage become enriched [2].

To date, the majority of insertional mutagenesis screens have utilized replicating gammaretroviruses (γRV) or transposons which have several limitations. Screens that use replicating retroviruses are limited to tissues and cell types that are permissive for replication of the virus. Because of this, the majority of screens have been performed in mouse hematopoietic cells or mouse mammary cells using replicating γRV vectors. Transposons allow mutagenesis of essentially any tissue and have expanded the use of mutagenesis screens. However, a major drawback of transposon approaches is the time it takes to generate the germline transgenic or knockout lines used, and to combine multiple alleles into the same background [3]. Another limitation of transposon mutagenesis is that multiple transposition events complicate the identification of causative mutagenic events [3].

One way to greatly expand the potential of forward mutagenesis screens is to use replication-incompetent lentiviral vectors (LVs) [4]. Human immunodeficiency virus (HIV)-derived LVs that are pseudotyped with the vesicular stomatitis virus glycoprotein can efficiently transduce essentially all mammalian cell types. Replication-incompetent LVs integrate into the genome but do not replicate, and thus do not create additional insertion sites. Therefore, there are fewer potential passenger integrations than with replication-competent vectors, where driver insertional mutation events may be masked by the accumulation of bystander integrations [4,5]. Also the level of mutagenesis can be carefully controlled by adjusting the multiplicity of infection. Importantly, under the right conditions replication-competent vectors can cause cancer, which was unfortunately observed in gene therapy studies where replication-incompetent γRV vectors caused leukemia [6,7].

Here we report a novel screen to identify genes in the progression to androgen-independent prostate cancer (AIPC) in human cells using a replication-incompetent HIV-based LV. Prostate cancer (PCa) is the second most common cause of cancer related deaths in men in the United States, and is one of the leading causes of sickness and death in men in the U.S. and Western Europe [8,9]. Despite this high prevalence, the molecular mechanisms of PCa progression still remain largely unknown, due in part to heterogeneity during tumor development [10]. PCa is initially androgen-dependent, making androgen deprivation therapy the first line of defense in combating the disease [11]. Though this treatment initially reduces tumor size, patients eventually develop AIPC, which is resistant to this primary form of therapy and is ultimately lethal [11-13]. Thus, determining the mechanisms that contribute to AIPC is critical to develop novel therapies for this advanced form of PCa. The current study was designed to identify genes involved in the progression to AIPC. The human LNCaP PCa cell line model for AIPC is well-established [11,14,15]. LNCaP cells express androgen receptor, prostate-specific antigen, and generate androgen-independence by distinct mechanisms [11,15]. Additionally, LNCaP cells readily form tumors in immunodeficient mice allowing *in vivo* studies [16,17]. A transposon-based mutagenesis screen has been performed for PCa that specifically investigated PCa precursor lesions and genes that were involved in PCa initiation, but AIPC mechanisms were not investigated [18].

A major advantage of our approach is the use of a LV shuttle vector that allows rescue of vector LTR-chromosome junctions in bacteria as plasmids. In other retroviral and transposon-based screens, PCR is typically used to recover these proviral insertions and in turn detect dysregulated genes [1,19]. However, PCR lacks the sensitivity to detect integrations events that are rare or poorly amplified. It has previously been suggested that plasmid-based rescue of the provirus integration might eventually replace PCR methods [1]. Our study demonstrates the potential of this approach in a mutagenesis screen for AIPC.

## Results

### Efficient LV transduction results in a library of mutagenized PCa cells where clonality can be rapidly assessed by shuttle vector rescue

**Figure 1:**
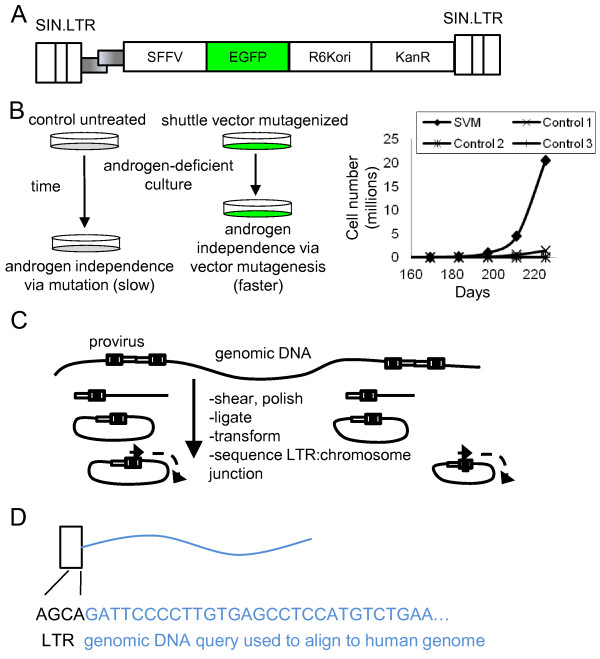
**LV-mediated mutagenesis screen. A)** Schematic of LV-SFFVEGFP vector. The strong spleen focus-forming viral (SFFV) promoter drives the expression of enhanced green fluorescent protein (EGFP). The vector includes a R6Kγ origin of replication (R6Kori) and kanamycin resistance gene (KanR) for rescue in *E.coli*. Both long terminal repeats are self-inactivating (SIN.LTR). **B)** Experimental outline for *in vitro* LV-mediated insertional mutagenesis screen. Mutagenized sample (SVM-A) became androgen-independent before control, non-mutagenized cells. At this time genomic DNA was extracted and analyzed by our shuttle vector rescue approach. **C)** Shuttle vector rescue. Genomic DNA is sheared into smaller fragments. The ends are polished and the fragments are ligated into plasmids. Plasmids are transformed into electrocompetent *E. coli*. Using an LTR-specific primer, the junction between the provirus LTR and the chromosome DNA is sequenced. **D)** To identify the proviral integration site the chromosomal DNA at the junction is used as a query and aligned to the human genome to identify the integration site.

To identify candidate genes involved in the progression to AIPC we used a replication-incompetent LV, LV-SFFVEGFP (Figure 1A) that has a strong spleen focus-forming virus promoter known to dysregulate genes [20], and also includes a bacterial origin of replication and a kanamycin resistance gene to allow identification of integration sites by rescue of shuttle vector plasmids in *E. coli*. Our approach uses random shearing of genomic DNA to avoid restriction site bias [21] and does not require any PCR amplification so it also eliminates PCR-based bias. The LV expresses EGFP gene from the spleen focus-forming virus promoter, allowing for efficient tracking of transduced cells *in vitro* and *in vivo* (Figure 1B). The androgen-dependent human PCa cell line, LNCaP, was transduced in triplicate with LV-SFFVEGFP resulting in three independent cultures of LNCaP cells denoted shuttle vector-mutagenized (SVM) -A, -B, and -C. The transduction frequency was over 99% as assessed by EGFP expression.

The clonality of these cultures prior to selection for androgen independence was evaluated to ensure that cells used for the mutagenesis screen were polyclonal. We reasoned that a highly polyclonal pool of integrations, in essence a library, would improve the ability of our screen to identify AIPC progression genes. High-throughput sequencing of shuttle vector rescued plasmids was performed to identify provirus-chromosome junctions (Figure 1C,D). Sequences were aligned to the human genome to identify the provirus integration site in the human genome. A clonality calculation was performed to approximate the number of unique integrations present in each sample prior to selection for androgen-independence. By counting both the number of new integration sites in each survey, and accounting for the number of previously identified integration sites, the approximate number of unique integration sites in a population could be calculated. A sample calculation for SVM-A is shown in Additional file 1: Figure S1. In this example, five surveys were performed and a clonality of 7.2 × 10^3^ unique integration sites was calculated. The more surveys that are performed, the larger N will become, improving the accuracy of the clonality estimate, so this is a minimum estimate. Clonality was similar for cultures SVM-B and SVM-C. Sequencing confirmed that there was no evidence of cross contamination between independent cultures, as no identical integration sites were identified between the three mutagenized cultures. These data showed that we had a highly polyclonal starting population prior to selection for androgen independence.

### *In vitro* screen to identify genes that influence androgen-independence

To model the clinical progression to advanced AIPC [22]*in vitro* we used a previously established method to select for cells that become androgen-independent in culture [11,14,15]. In this model androgen-dependent LNCaP cells are cultured in charcoal/dextran-treated fetal bovine serum (CT-FBS) which is essentially devoid of androgen. This selects for those cell clones that have a proliferative advantage in an androgen-deficient environment (Figure 1B). The human LNCaP cell line has several advantages for our screen including expression of androgen receptor, androgen-dependent growth, a demonstrated ability to develop androgen-independent growth [11,14,15], and the ability to form tumors *in vivo*[16,23] to allow us to explore genes and gene pathways that mediate progression *in vivo*. Preliminary experiments showed that transfer of LNCaP cells into media supplemented with 10% CT-FBS led to a loss of cells which would have reduced the clonality of our LV-mutagenized library of insertion sites. We found that initial culture in 9.75% CT-FBS with 0.25% untreated FBS minimized cell loss. We thus cultured LV-mutagenized and control cells in media supplemented with 9.75% CT-FBS with 0.25% untreated FBS for approximately 140 days prior to moving cultures to media supplemented with 10% CT-FBS. At this time shuttle-vector mutagenized androgen-dependent LNCaP cells were maintained in an androgen-deficient environment with CT-FBS. After 211 days, the SVM-A culture showed an increase in growth rate compared to control cultures, and was deemed androgen-independent (Figure 1B). We hypothesized that in the SVM-A culture, cells with LV proviruses near genes that influenced progression to AIPC had a selective advantage and this led to androgen-independent growth in the SVM-A culture prior to control cultures. This is expected to lead to an over-representation of cells with proviral integrants near genes that influence AIPC. Thus, analysis of provirus integration sites in these androgen-independent cells should identify dysregulated genes near vector proviruses that may mediate progression to AIPC. Genomic DNA was isolated from LV-transduced androgen-independent cells to identify candidate AIPC progression genes by shuttle vector rescue (Figure 1C).

### *In vivo* xenotransplant approach to identify genes that influence androgen-independence

**Figure 2:**
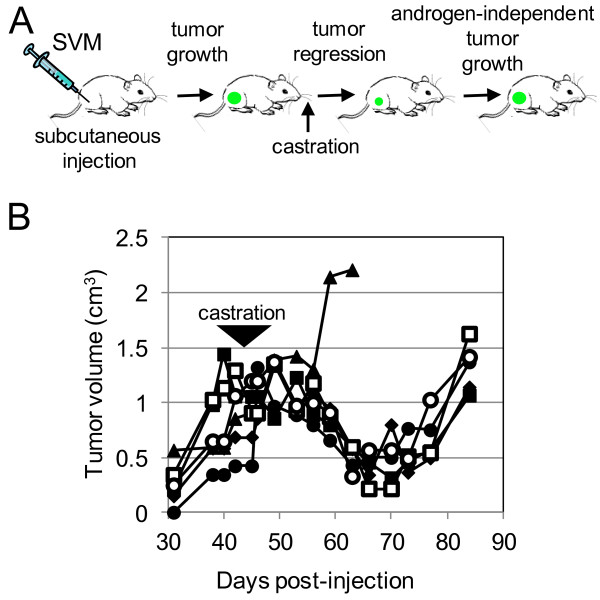
***In vivo *LV-mediated insertional mutagenesis screen. A)***In vivo* approach. Mice were injected with shuttle-vector mutagenized LNCaPs subcutaneously in the right flank. Following tumor development, mice were castrated and the tumors regressed. Following castration tumors regressed in most mice. When the tumor re-grew to larger than pre-castration size, the tumor was removed, genomic DNA was extracted, and shuttle vector rescue was performed. **B)** Tumor growth *in vivo*. Animals were castrated between days 40-50 as indicated by the arrow.

*In vitro* models lack the ability to identify genes involved in processes required only *in vivo* such as vascularization. We thus also performed our LV shuttle vector screen *in vivo* using a LNCaP xenograft model [16,23]. NOD.Cg-PrkdcscidIl2rgtmlWjl/SzJ (NSG) mice are severely immunocompromised, and allow for efficient engraftment of human cells [24]. Androgen-dependent control LNCaPs and mutagenized SVM-A cells prior to selection *in vitro* were injected into male NSG mice (Figure 2A). Tumors developed from the injection of both SVM-A and control LNCaP cells. Similar to androgen-dependent tumors in PCa patients, it was expected that tumor volumes would regress following androgen deprivation therapy. In patients, this is done by either a surgical or chemical castration [22]. In the *in vivo* model, this environment was created by surgical removal of the testes which are the primary source of androgens. It was expected that tumor size would decrease immediately following castration and similar to the *in vitro* model, the androgen-deficient environment would select for androgen-independent cells modeling what occurs in PCa patients [11,12]. LNCaP cells were injected in male NSG mice and formed in 6 of 7 injected mice (Figure 2B). Tumors did regress following castration and following the castration, tumor growth resumed. Tumors were allowed to grow until they reached volumes larger than the tumor size prior to castration, at which point tumors were harvested and genomic DNA was obtained for shuttle vector rescue analysis.

### Identification of genes near LV integration sites isolated from *in vitro* androgen-independent cultures and subcutaneous tumors from castrated mice

Rescue of genomic DNA from the androgen-independent culture SVM-A *in vitro* samples post-selection recovered a total of 21 unique sites (Additional file 2: Table S1). From two *in vivo* tumors analyzed post-castration we identified a total of 54 insertion sites, 27 from one tumor and 28 from the second with one site found in both tumors (Additional file 2: Table S1). All sites were single insertion sites. Custom PERL computer programs were used to analyze whether the provirus integrated within a RefSeq gene and to also provide the distance from the integration site to the nearest 3 Refseq gene transcription start sites (TSS). Only genes that had a TSS within 100 kb of the vector provirus were considered.

### Enrichment of vector proviruses within PCa susceptibility loci after selection for androgen-independence

**Figure 3:**
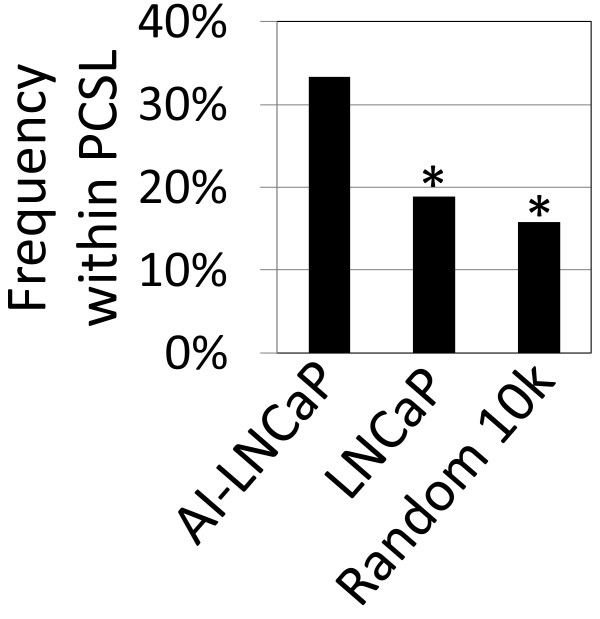
**LV integrants are enriched in PCa susceptibility loci after selection for androgen-independence.** Lentiviral integration sites (75) identified from LNCaP androgen-independent cultures *in vitro* or tumors from castrated animals (AI-LNCaP) were mapped relative to previously described PCa susceptibility loci (PCSL). The frequency of these sites was compared to LNCaP cells pre-selection (LNCaP) and to a control data set of 10,000 random sites. *indicates significant difference of p < 0.001.

We hypothesized that our approach should enrich for vector integration sites near regions known to be involved in PCa progression. The 75 integration sites (Additional file 2: Table S1) were mapped relative to previously described PCa susceptibility loci (Additional file 3: Table S2) and compared to 412 control integration sites obtained from transduced LNCaP cells prior to selection for androgen-independence, and also to a random *in silico* generated data set of 10,000 sites (Figure 3). The percent of vector integrations within PCa susceptibility loci for LNCaP cells cultured in androgen-deficient medium was significantly higher than from the LNCaP cells prior to culture in androgen-deficient culture medium (p < .001) and also significantly higher than from the random sites (p < .001). This demonstrates a significant enrichment of vector proviruses near loci previously associated with PCa when cells are grown under androgen-deficient conditions.

### Meta-analysis of expression of candidate PCa genes near vector proviruses

**Figure 4:**
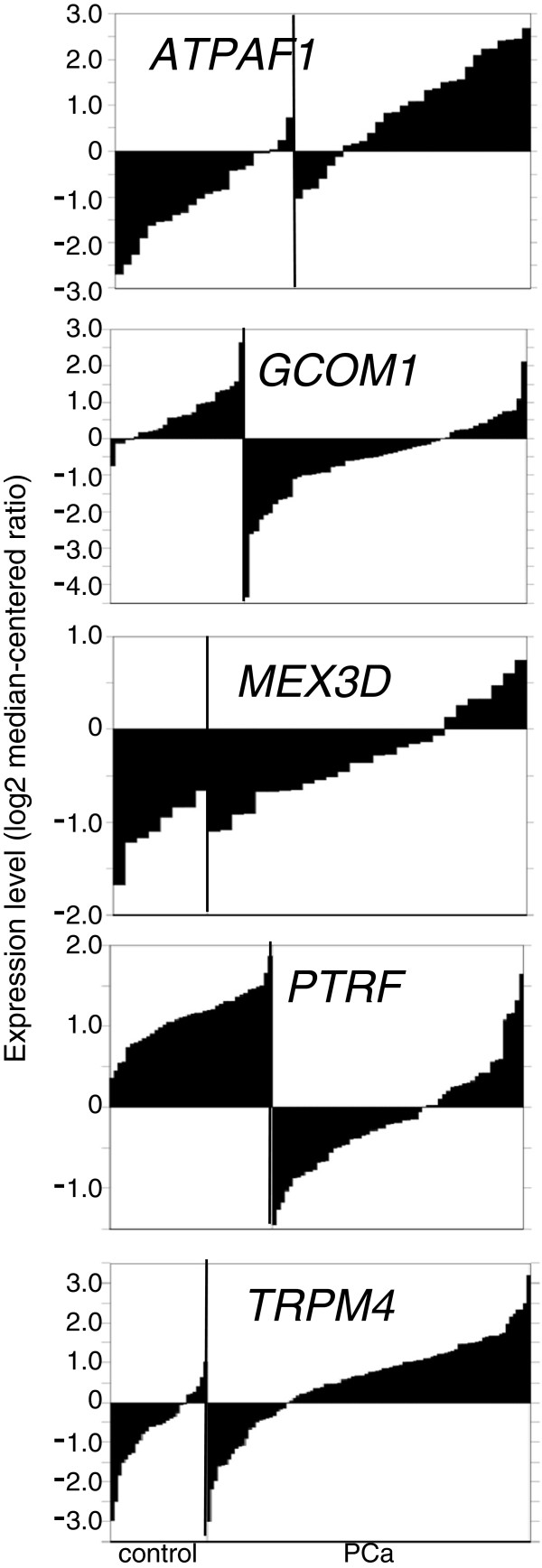
**Differential expression in normal prostate tissue vs. PCa tissue for candidate AIPC genes.** Oncomine™ (Compendia Bioscience, Ann Arbor, MI) was used for analysis and visualization. The Oncomine™ database was queried using gene names for *ATPAF1, GCOM1, MEX3D, PTRF*, and *TRPM4* using the Cancer vs. Normal Analysis, and selecting PCa vs. Normal analysis. This provides gene expression levels in healthy prostate tissue (left side, control), and prostate carcinoma or prostate adenocarcinoma tissue samples (right side, PCa). The data is represented here as waterfall plots for each different gene.

We used data from previously published microarray analysis of patient tumors to identify the genes in our dataset that were most likely to contribute to AIPC based on dysregulation in patient tumors. This approach has the advantage that multiple data sources may improve the power of the screen, and may improve the ability to identify genes that are clinically relevant due to their dysregulation in patient tissues. We used Oncomine™ (Compendia Bioscience, Ann Arbor, MI) [25] to compare microarray data from 16 different studies [26-39] that evaluated gene expression in normal prostate tissue vs. PCa tissue (prostate carcinoma or prostate adenocarcinoma) to evaluate the candidate progression genes identified near vector proviruses listed in Additional file 2: Table S1. We reasoned that candidate driver genes identified in our screen were likely to be over or under-expressed in PCa tissue and that meta-analysis of previously published microarray studies would be a powerful way to screen our candidates for driver genes likely to be potential biomarkers or therapeutic targets. To evaluate over or under-expression we used both the rank and a multiple comparisons-corrected p-value as calculated by Oncomine™. Candidate genes with p-values < .005 and ranks less than 1000 in either the over- or under-expression multiple comparisons were considered as potential driver genes (Table 1). Promising candidates identified from the androgen-independent *in vitro* culture were *ATPAF1* (ATP synthase mitochondrial F1 complex assembly factor 1) and *TRPM4* (transient receptor potential cation channel, subfamily M, member 4). Promising candidates identified from the androgen-independent *in vivo* tumor were *GCOM1* (GRINL1A complex locus 1)*, MEX3D* (mex-3 RNA binding family member D), and *PTRF* (polymerase I and transcript release factor). Three of these genes, *ATPAF1*, *MEX3D* and *TRPM4,* are over-expressed in PCa tissue, while two are under-expressed, *GCOM1* and *PTRF* (Figure 4 and Additional file 4: Figure S2). The locations of the five vector integrants were mapped relative to genomic loci using the University of California, Santa Cruz (UCSC) Genome Browser (Additional file 5: Figure S3 and Additional file 6: Figure S4).

**Table 1:** Candidate AIPC genes

**Gene**	**Source**	**In gene/distance**	**PCSL**	**Expression**	**p-value**
*ATPAF1*	*In vitro*	yes	none	over	0.002
*GCOM1*	*In vivo*	yes	none	under	0.003
*MEX3D*	*In vivo*	32,561	19p13.3	over	9.70E-04
*PTRF*	*In vivo*	62,474	17q21-22	under	0.003
*TRPM4*	*In vitro*	yes	near 19q13.4	over	1.13E-04

Candidate AIPC genes identified from provirus integration sites recovered from both the *in vitro* mutagenesis screen and the *in vivo* subcutaneous tumor model. Source indicates whether the gene was identified from the *in vitro* screen or the *in vivo* screen. In gene/distance indicates whether the LV provirus was within the gene transcription unit, or if outside the transcription unit indicates the distance in base pairs from the proviral integration to the transcription start site of the gene. PCSL indicates if the provirus was within or near a PCa susceptibility locus. Expression indicates if the gene was over or under-expressed in PCa tissue using the Oncomine™ database. Rank is the Oncomine™ ranking of over or under-expressed genes. p-value is the Oncomine™ p-value for over or under-expression.

### Validation of genes involved in AIPC by RNAi knockdown confirms involvement of *ATPAF1* and in PCa progression

**Figure 5:**
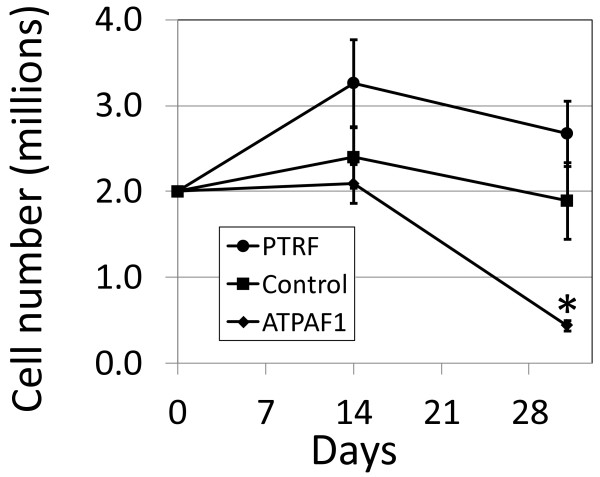
**Validation using LV-mediated knockdown.** LNCaP cells were transduced with a pGIPZ lentiviral vector expressing a shRNA targeting either *ATPAF1*, *PTRF*, or a control empty vector. Vector-exposed cells were selected using puromycin to eliminate untransduced cells. Transduced cells were then cultured in androgen-deficient medium to determine if knockdown of *ATPAF1* or *PTRF* affected growth.

To validate the effects of *ATPAF1* and *PTRF* on androgen-independence we used LV-mediated RNAi*.* For *ATPAF1* and *PTRF* a set of commercially available pGIPZ LV vectors targeting different sites on each gene were first screened to find an efficient shRNA target (Additional file 7: Figure S5). The pGIPZ vector with the most effective shRNA was then used to transduce LNCaP cells. Cells were then cultured with puromycin to eliminate untransduced cells. Knockdown was confirmed by RT-PCR (Additional file 7: Figure S5C**)** and then puromycin-resistant cells were cultured under androgen-deficient conditions to select for androgen-independence. *PTRF* is under-expressed in PCa tissue, so *PTRF* knockdown was expected to lead to more rapid proliferation under androgen-deficient conditions*.* Conversely, *ATPAF1* is overexpressed in PCa tissue, so knockdown of this gene should impair proliferation under androgen-deficient conditions. After 31 days of culture in androgen-deficient medium, as expected knockdown of *PTRF* resulted in higher cell numbers than cells transduced with the control vector, however the increase did not reach statistical significance (p = 0.25) (Figure 5). Knockdown of *ATPAF1* resulted in significantly fewer cells in androgen-deficient medium than the control by day 31 of culture (p < 0.05).

## Discussion

PCa remains a significant health problem and a leading cause of cancer related death in the United States and parts of Europe [8,9]. However, many of PCa’s molecular mechanisms remain elusive. Though treatable in its early, androgen-dependent stages, PCa often progresses to a lethal, untreatable, androgen-independent form. Here, we report for the first time, a retroviral mutagenesis screen using a replication-incompetent LV vector to identify genes that mediate progression to AIPC. Our shuttle vector approach efficiently recovered integration sites that could be rapidly mapped to genomic loci. We observed a strong enrichment of provirus integrations in PCa susceptibility loci when integration sites were compared between transduced cells prior to culture in androgen-deficient medium and after cells had become androgen-independent. This suggests that integrated vector proviruses dysregulated nearby genes that allowed these clones to proliferate in the absence of androgen, and provided a selective advantage resulting in their enrichment in a polyclonal population. While this has been widely exploited for retrovirus mutagenesis screens, we show here for the first time that a replication-incompetent LV can be used to identify candidate genes that mediate AIPC. By performing meta-analysis of our data with patient data we identified 5 promising candidate AIPC genes from 75 unique integration sites.

**Figure 6:**
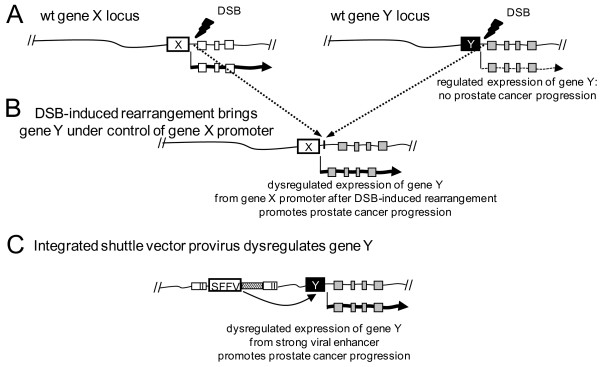
**Shuttle vector mutagenesis models a major mechanism whereby DSB-induced rearrangements dysregulate genes.** In panel **A** two loci are shown where a strong promoter expresses gene X and a weaker regulated promoter expresses gene Y. In panel **B** a DSB-induced rearrangement places gene Y under the control of the gene X promoter, dysregulating gene Y and thereby promoting PCa progression. In panel **C** an integrated shuttle vector provirus enhances expression of gene Y promoting PCa progression.

High-throughput gene expression studies [40-42], comparative genome hybridization studies [43,44], and proteome studies [45-47] have previously been used to identify PCa genes. However differentiating driver genes from passenger genes using these approaches has been difficult. Retroviral mutagenesis identifies driver genes by dysregulating genes near vector proviruses that provide a selective advantage. It also models a major mechanism for PCa, the formation of double-strand breaks (DSBs) (Figure 6) which can result in gene fusions such as TMPRSS-ERG [48,49]. These gene fusions often place constitutively active promoters next to oncogenes, resulting in activation of an oncogene. LV-based mutagenesis can model mutations caused by DSBs as we use a strong viral promoter/enhancer to dysregulate neighboring genes, analogous to the juxtaposition of strong promoters to nearby proto-oncogenes (Figure 6C).

Using a replication-incompetent LV shuttle vector has several advantages. LVs can be produced at high titer and the vesicular stomatitis virus glycoprotein used to pseudotype the LV has a broad tropism allowing for efficient transduction of essentially any target mammalian cell. Thus our approach can be used to identify genes involved in numerous oncogenic processes. For example, such a screen could be used to identify genes involved in the progression to metastatic breast cancer. Further, the approach can be used to identify genes involved in virtually any biological process that involves cells that undergo a selective pressure. A novel advantage of our approach is in the method of recovery of the provirus integration site. Previous mutagenesis screens have used PCR to detect proviral integrations but PCR is limited in its ability to identify rare or poorly amplified insertions [1]. Additionally, PCR sequence read lengths are shorter than those recovered using a shuttle vector rescue technology [21]. The longer sequence reads provided by shuttle vector allow for a more accurate localization of the provirus integration. There are some limitations to the design of our mutagenesis screen. The LV used contains self-inactivating (SIN) LTRs with modified U3 region in the 3′ long-terminal-repeat (LTR) sequences and with deleted viral enhancers. The lack of transcriptionally active LTRs in SIN LVs reduces genotoxicity relative to lentiviral or gammaretroviral vectors with an intact LTR [20]. Future studies will explore the effect of retroviral vector type and design on the mutagenic potential.

Our screen identified five candidate PCa progression genes; *ATPAF1, GCOM1, MEX3D, PTRF,* and *TRPM4. TRPM4* has been associated with the development of a cancer phenotype, particularly with cell proliferation and tumor progression [50,51], and has been specifically shown to enhance cell proliferation through up-regulation of the β-catenin signaling pathway [50,52]. *GCOM1* is a locus with a naturally occurring read-through transcription event, with one transcript encoding a fusion protein [53]. Interestingly *GCOM1* was identified as an estrogen receptor β (ERβ) target gene [54]. Estrogen receptors appear to play an important role in PCa and ERβ is expressed in the prostate gland [55]. ERβ is the main target for phytoestrogens, and may play a role in the difference in incidence of PCa in the Western world compared to Asia where the intake of soy-based, phytoestrogen-rich food is higher [55]. *PTRF* has been associated with a number of different functions in the prostate and in cancer. In PCa, *PTRF* expression has been shown to alter the aggressiveness of the cancer [56]. *PTRF* has also been shown to be decreased significantly in LNCaP and PC3 cells and in cancer tissue [57]. *PTRF* is involved in the formation of caveolae, invaginations of the plasma membrane [58,59]. *PTRF* is also involved in localization of caveolin-1, which is involved in PCa severity, aggressiveness, metastasis, and androgen sensitivity [58]. Additionally, *PTRF* is under-expressed in PCa, and its expression can actually attenuate PCa disease severity and aggressiveness [58].

While these genes have been previously associated with cancer or prostate physiology, our screen was also able to identify novel candidate PCa progression genes. *MEX3D*, is a member of the Mex-3 subfamily of conserved RNA-binding proteins, which are involved in post-transcriptional regulation [60]. *MEX3D* was previously associated with chemotherapy-induced oral mucositis in acute myeloid leukemia patients [61]. *ATPAF1*, another novel target, encodes an assembly factor for the F1 component of the mitochondrial ATP synthase, which is required for assembly of the ATP synthase F_1_ complex in oxidative phosphorylation [62]. *ATPAF1* is widely expressed in host tissues, but to our knowledge has never been specifically linked to cancer. However, another mitochondrial protein involved in mitochondrial oxidative phosphorylation, ATPase inhibitory factor 1 (*ATPIF1*) mediates the metabolic shift of cancer cells to a Warburg phenotype and has been identified as a promising predictive marker for clinical outcome in breast and colon cancer [63].

Our data suggests an involvement of both *PTRF* and *ATPAF1* in AIPC. *ATPAF1* knockdown resulted in significantly decreased growth relative to controls, however the increase in growth was not significant for *PTRF* knockdown. *PTRF* expression is decreased in PCa cells, including LNCaP and PC-3 [57]. We speculate that because the level of *PTRF* expression in LNCaP cells is normally low, that any effect from knockdown by RNAi is limited. Clearly further experiments are warranted to confirm the role of PTRF and *ATPAF1* in AIPC, but our study is the first to our knowledge to implicate *ATPAF1* in AIPC.

## Conclusions

We utilized a replication-incompetent shuttle LV as an insertional mutagen to identify genes involved in the progression to AIPC. This approach has proved effective for use in PCa, showing enrichment of proviral integrations near PCa susceptibility loci and identifying 5 potential driver genes, 1 of which was validated. Our approach has broad potential applications for cancer gene discovery, and for interrogating diverse biological and disease processes.

## Methods

### Cell line culture, vector production and transduction

The androgen-dependent human prostate carcinoma cell line LNCaP-FGC (ATCC CRL-1740) was cultured in RPMI-1640 supplemented with 10% FBS (Atlanta Biologicals, Lawrenceville, GA) at 37°C in 5% CO_2_. The LV shuttle vector, LV-SFFVEGFP, has self-inactivating long terminal repeats (LTRs), an internal spleen focus-forming virus promoter driving EGFP expression, and R6Kγ origin of replication and a neomycin phosphotransferase gene. Vesicular stomatitis virus glycoprotein pseudotyped vector stocks were made by PEI-mediated transfection of HEK-293T cells as previously described [64]. Functional titers were determined by transduction of HT-1080 fibrosarcoma cells. Cells were cultured for 14 days post vector exposure prior to use in experiments.

### Shuttle vector rescue in bacteria, identification of integration sites and clonality evaluation

Genomic DNA was isolated using the Puregene Cell & Tissue kit (Qiagen Inc., Valencia, CA) and was sheared using a Hydroshear (DigiLab Inc., Marlborough, MA). The ends of the DNA were repaired using the Terminator End Repair Kit (Lucigen Corporation, Middleton, WI). Sheared fragments were ligated using T4 DNA Ligase (New England Biolabs, Inc., Ipswich, MA) and transformed by electroporation. Shuttle vector plasmids from kanamycin-resistant colonies were sequenced using primers specific to the LV LTR. A total of 288 colonies were sequenced for the *in vitro* cultures and 96 colonies were sequenced for each tumor. The junction between the integrated provirus and the chromosome was identified and integration sites in the human genome (hg19) were determined using PERL bioinformatics programs [21] that invoke a standalone BLAT program [65]. To be considered as an integration site the alignment score had to have a canonical LTR-chromosome junction and meet additional strict criteria as previously described [21]. A control dataset of 10,000 random sites was generated as previously described [65]. The Schnabel method of multiple-census mark-recapture was used to approximate the number of unique integration sites in the entire population prior to selection for androgen independence [66,67].

### *In vitro* androgen-independent culture

Control and LV-SFFVEGFP transduced LNCaP cells were maintained in RPMI-1640 supplemented with 9.75% charcoal/dextran-treated FBS (CT-FBS) and 0.25% FBS (Atlanta Biologicals, Lawrenceville, GA), before being moved to and maintained in RPMI-1640 supplemented with 10% CT-FBS. Cells were counted using a Cellometer Auto T4 (Nexcelom Bioscience Inc., Lawrence, MA) and replated approximately every 2-3 weeks.

### *In vivo* model of PCa

All protocols involving the use of animals were approved by the Washington State University Institutional Animal Care and Use Committee and institutional guidelines for the humane use of animals in research were followed. Male 4-8 week old NSG mice were obtained from The Jackson Laboratory (Bar Harbor, Maine). LV-mutagenized or control LNCaP cells were inoculated via subcutaneous injection. Between 1-5×10^6^ cells were suspended in 100 μL of RPMI-1640 plus 5% FBS plus 100 μL Matrigel (BD Biosciences, Bedford, MA) and injected via a 25 gauge needle into the right flank. Tumors were visually monitored or measured once to twice weekly and their volumes were calculated using the formula L×W×H×0.5236 [17].

To select for androgen-independent tumor growth *in vivo*, mice were castrated via the scrotal approach. Mice were anesthetized with isoflurane or ketamine and xylazine (80mg/kg and 6mg/kg, respectively). When tumors reached volumes larger than the tumor size prior to castration, mice were sacrificed, tumor tissue was harvested. Genomic DNA was obtained from tumor tissue using the Puregene Cell & Tissue kit (Qiagen Inc., Valencia, CA).

### Validation of the effect of target genes on AIPC using LV-mediated RNAi

pGIPZ lentiviral shRNA vector sets of 3-6 shRNA vectors were obtained from Thermo Fisher Scientific (Waltham, MA) for the selected target genes. Gene knockdown was evaluated using the psiCHECK™-2 system (Promega Corporation, Madison, WI) using a synthesized sequence (GenScript USA, Inc., Piscataway, NJ) containing target sites for each target gene shRNA cloned into the psiCHECK™-2 vector. psiCHECK™-2 vectors containing these target fragments were co-transfected with pGIPZ shRNA vector sets for each specific target gene. The efficiency of the knockdown for each shRNA for each gene was determined with the Dual-Luciferase® Reporter Assay System (Promega Corporation, Madison, WI). The shRNA which demonstrated the most efficient knockdown of luciferase activity for each target gene was selected. Vesicular stomatitis virus glycoprotein pseudotyped vector stocks were made by PEI-mediated transfection of HEK-293T cells as described above. LNCaP cells for *in vitro* validation were transduced at a multiplicity of infection of 5, selected in 2.5 μg/mL puromycin, and expanded. RT-PCR was used to quantitate the expression levels of these genes in the pGIPZ transduced LNCaP cells. Total RNA from LNCaP cells transduced with a LV carrying a shRNA sequence against the candidate genes or vector with no shRNA (EV) was isolated using TRIzol reagent (Invitrogen, Carlsbad, CA). cDNA was synthesized from the total RNA using Transcription First Strand Synthesis Kit (Roche Diagnostics, Indianapolis, IN). cDNA was used as a template to amplify gene specific products using primers *ATPAF1* (forward: 5′- GCCAACCAAGTTCAGCTCTT-3′ and reverse 5′-GGTTCTGGGCACATTTCAGT-3′), *PTRF* (forward: 5′-GAAGAGCTGATCAAGTCGGACC-3′ and reverse: 5′-GCTTCACTTCATCCTGGTAGATCA-3′) and Glyceraldehyde-3-phosphate dehydrogenase (GAPDH; forward: 5′-ATCCCATCACCATCTTCCAG-3′ and reverse: 5′- CCATCACGCCACAGTTTCC-3′). Transduced puromycin-resistant LNCaP cells were cultured in androgen-deficient medium and cell number determined as described above.

### Statistical analysis

To assess the enrichment of proviruses in PCa susceptibility loci after selection for androgen-independence, a χ^2^ test of the frequency of integration sites was used. To assess the effects of shRNA knockdown of *PTRF* and *ATPAF1* on androgen-independence a Student’s t-test was used.

## Abbreviations

γRV: Gammaretrovirus; LV: Lentiviral vector; AIPC: Androgen-independent prostate cancer; PCa: Prostate cancer; SFFV: Spleen focus-forming viral promoter; EGFP: Enhanced green fluorescent protein; LTR: Long terminal repeat; SVM: Shuttle vector mutagenized; PCSL: Prostate cancer susceptibility loci.

## Competing interests

The authors indicated no potential competing interests.

## Authors’ contributions

ENS carried out the *in vitro* and *in vivo* screens, performed validation, analyzed data and drafted the manuscript. VB performed validation. AN performed the RT-PCR analysis for the validation. DTR carried out vector construction. LT generated viral vector preparations and mutagenized the LNCaP cells. GGM assisted with the study design and edited the manuscript. GDT conceived the study, drafted the manuscript and analyzed data. All authors read and approved the final manuscript.

## Supplementary Material

Additional file 1: Figure S1 Determining the clonality of LV integrants. Click here for file

Additional file 2: Table S1 Proviral integration sites identified from both in vitro and in vivo androgen-independent samples. The in vitro androgen-independent culture SVM-A led to the identification of 21 unique integration sites. The in vivo androgen-independent subcutaneous (s.c.) tumors led to the identification of 54 unique integration sites. In total, 75 unique integration sites were identified. Click here for file

Additional file 3: Table S2 PCa susceptibility loci. Click here for file

Additional file 4: Figure S2 Oncomine meta-analysis of expression in prostate cancer tissue of genes near vector proviruses. Click here for file

Additional file 5: Figure S3 Localization of vector proviruses to the human genome. Click here for file

Additional file 6: Figure S4 Proviral integration sites displayed using the UCSC genome browser. The positions of the proviral integration site is indicated by the vertical line on images generated by the University of California, Santa Cruz genome browser. Click here for file

Additional file 7: Figure S5 Screening of pGIPZ knockdown vectors for ATPAF1 and PTRF and confirmation of knockdown by RT-PCR. Click here for file

## References

[B1] Uren AG Kool J Berns A van Lohuizen M Retroviral insertional mutagenesis: past, present and future Oncogene 2005 24 7656 7672 10.1038/sj.onc.1209043 16299527

[B2] Trobridge GD Genotoxicity of retroviral hematopoietic stem cell gene therapy Expert Opin Biol Ther 2011 11 581 593 10.1517/14712598.2011.562496 21375467 PMC3443588

[B3] Landrette SF Xu T Somatic genetics empowers the mouse for modeling and interrogating developmental and disease processes PLoS Genet 2011 7 e1002110 10.1371/journal.pgen.1002110 21814514 PMC3140981

[B4] Ranzani M Cesana D Bartholomae CC Sanvito F Pala M Benedicenti F Gallina P Sergi LS Merella S Bulfone A Doglioni C von Kalle C Kim YJ Schmidt M Tonon G Naldini L Montini E Lentiviral vector-based insertional mutagenesis identifies genes associated with liver cancer Nat Methods 2013 10 155 161 10.1038/nmeth.2331 23314173 PMC3589714

[B5] Collier LS Carlson CM Ravimohan S Dupuy AJ Largaespada DA Cancer gene discovery in solid tumours using transposon-based somatic mutagenesis in the mouse Nature 2005 436 272 276 10.1038/nature03681 16015333

[B6] Hacein-Bey-Abina S Garrigue A Wang GP Soulier J Lim A Morillon E Clappier E Caccavelli L Delabesse E Beldjord K Asnafi V MacIntyre E Dal Cortivo L Radford I Brousse N Sigaux F Moshous D Hauer J Borkhardt A Belohradsky BH Wintergerst U Velez MC Leiva L Sorensen R Wulffraat N Blanche S Bushman FD Fischer A Cavazzana-Calvo M Insertional oncogenesis in 4 patients after retrovirus-mediated gene therapy of SCID-X1 J Clin Invest 2008 118 3132 3142 10.1172/JCI35700 18688285 PMC2496963

[B7] Hacein-Bey-Abina S Von Kalle C Schmidt M McCormack MP Wulffraat N Leboulch P Lim A Osborne CS Pawliuk R Morillon E Sorensen R Forster A Fraser P Cohen JI de Saint Basile G Alexander I Wintergerst U Frebourg T Aurias A Stoppa-Lyonnet D Romana S Radford-Weiss I Gross F Valensi F Delabesse E Macintyre E Sigaux F Soulier J Leiva LE Wissler M LMO2-associated clonal T cell proliferation in two patients after gene therapy for SCID-X1 Science 2003 302 415 419 10.1126/science.1088547 14564000

[B8] Visakorpi T The molecular genetics of prostate cancer Urology 2003 62 3 10 14607212 10.1016/s0090-4295(03)00776-3

[B9] Nelson WG De Marzo AM Isaacs WB Prostate cancer N Engl J Med 2003 349 366 381 10.1056/NEJMra021562 12878745

[B10] Porkka KP Visakorpi T Molecular mechanisms of prostate cancer Eur Urol 2004 45 683 691 10.1016/j.eururo.2004.01.012 15149739

[B11] Lu S Tsai SY Tsai MJ Molecular mechanisms of androgen-independent growth of human prostate cancer LNCaP-AI cells Endocrinology 1999 140 5054 5059 10537131 10.1210/endo.140.11.7086

[B12] Gulley J Figg WD Dahut WL Treatment options for androgen-independent prostate cancer Clin Adv Hematol Oncol 2003 1 49 57 16227960

[B13] Marech I Vacca A Ranieri G Gnoni A Dammacco F Novel strategies in the treatment of castration-resistant prostate cancer (Review) Int J Oncol 2012 40 1313 1320 22322981 10.3892/ijo.2012.1364

[B14] Pousette A Carlstrom K Henriksson P Grande M Stege R Use of a hormone-sensitive (LNCaP) and a hormone-resistant (LNCaP-r) cell line in prostate cancer research Prostate 1997 31 198 203 10.1002/(SICI)1097-0045(19970515)31:3<198::AID-PROS9>3.0.CO;2-H 9167773

[B15] Iguchi K Ishii K Nakano T Otsuka T Usui S Sugimura Y Hirano K Isolation and characterization of LNCaP sublines differing in hormone sensitivity J Androl 2007 28 670 678 10.2164/jandrol.107.002675 17409465

[B16] Horoszewicz JS Leong SS Kawinski E Karr JP Rosenthal H Chu TM Mirand EA Murphy GP LNCaP model of human prostatic carcinoma Cancer Res 1983 43 1809 1818 6831420

[B17] Sato N Gleave ME Bruchovsky N Rennie PS Beraldi E Sullivan LD A metastatic and androgen-sensitive human prostate cancer model using intraprostatic inoculation of LNCaP cells in SCID mice Cancer Res 1997 57 1584 1589 9108464

[B18] Rahrmann EP Collier LS Knutson TP Doyal ME Kuslak SL Green LE Malinowski RL Roethe L Akagi K Waknitz M Huang W Largaespada DA Marker PC Identification of PDE4D as a proliferation promoting factor in prostate cancer using a Sleeping Beauty transposon-based somatic mutagenesis screen Cancer Res 2009 69 4388 4397 10.1158/0008-5472.CAN-08-3901 19401450 PMC2710962

[B19] Heim D Cornils K Schulze K Fehse B Lohse AW Brummendorf TH Wege H Retroviral insertional mutagenesis in telomerase-immortalized hepatocytes identifies RIPK4 as novel tumor suppressor in human hepatocarcinogenesis Oncogene 2014 doi:10.1038/onc.2013.551 10.1038/onc.2013.551 24413083

[B20] Montini E Cesana D Schmidt M Sanvito F Bartholomae CC Ranzani M Benedicenti F Sergi LS Ambrosi A Ponzoni M Doglioni C Di Serio C von Kalle C Naldini L The genotoxic potential of retroviral vectors is strongly modulated by vector design and integration site selection in a mouse model of HSC gene therapy J Clin Invest 2009 119 964 975 10.1172/JCI37630 19307726 PMC2662564

[B21] Trobridge GD Miller DG Jacobs MA Allen JM Kiem HP Kaul R Russell DW Foamy virus vector integration sites in normal human cells Proc Natl Acad Sci U S A 2006 103 1498 1503 10.1073/pnas.0510046103 16428288 PMC1360565

[B22] Sharifi N Gulley JL Dahut WL Androgen deprivation therapy for prostate cancer Jama 2005 294 238 244 10.1001/jama.294.2.238 16014598

[B23] Wang X An Z Geller J Hoffman RM High-malignancy orthotopic nude mouse model of human prostate cancer LNCaP Prostate 1999 39 182 186 10.1002/(SICI)1097-0045(19990515)39:3<182::AID-PROS6>3.0.CO;2-B 10334107

[B24] Shultz LD Lyons BL Burzenski LM Gott B Chen X Chaleff S Kotb M Gillies SD King M Mangada J Greiner DL Handgretinger R Human lymphoid and myeloid cell development in NOD/LtSz-scid IL2R gamma null mice engrafted with mobilized human hemopoietic stem cells J Immunol 2005 174 6477 6489 10.4049/jimmunol.174.10.6477 15879151

[B25] Rhodes DR Yu J Shanker K Deshpande N Varambally R Ghosh D Barrette T Pandey A Chinnaiyan AM ONCOMINE: a cancer microarray database and integrated data-mining platform Neoplasia 2004 6 1 6 10.1016/S1476-5586(04)80047-2 15068665 PMC1635162

[B26] Arredouani MS Lu B Bhasin M Eljanne M Yue W Mosquera JM Bubley GJ Li V Rubin MA Libermann TA Sanda MG Identification of the transcription factor single-minded homologue 2 as a potential biomarker and immunotherapy target in prostate cancer Clin Cancer Res 2009 15 5794 5802 10.1158/1078-0432.CCR-09-0911 19737960 PMC5573151

[B27] Grasso CS Wu YM Robinson DR Cao X Dhanasekaran SM Khan AP Quist MJ Jing X Lonigro RJ Brenner JC Asangani IA Ateeq B Chun SY Siddiqui J Sam L Anstett M Mehra R Prensner JR Palanisamy N Ryslik GA Vandin F Raphael BJ Kunju LP Rhodes DR Pienta KJ Chinnaiyan AM Tomlins SA The mutational landscape of lethal castration-resistant prostate cancer Nature 2012 487 239 243 10.1038/nature11125 22722839 PMC3396711

[B28] Holzbeierlein J Lal P LaTulippe E Smith A Satagopan J Zhang L Ryan C Smith S Scher H Scardino P Reuter V Gerald WL Gene expression analysis of human prostate carcinoma during hormonal therapy identifies androgen-responsive genes and mechanisms of therapy resistance Am J Pathol 2004 164 217 227 10.1016/S0002-9440(10)63112-4 14695335 PMC1602218

[B29] Lapointe J Li C Higgins JP van de Rijn M Bair E Montgomery K Ferrari M Egevad L Rayford W Bergerheim U Ekman P DeMarzo AM Tibshirani R Botstein D Brown PO Brooks JD Pollack JR Gene expression profiling identifies clinically relevant subtypes of prostate cancer Proc Natl Acad Sci U S A 2004 101 811 816 10.1073/pnas.0304146101 14711987 PMC321763

[B30] Liu P Ramachandran S Ali Seyed M Scharer CD Laycock N Dalton WB Williams H Karanam S Datta MW Jaye DL Moreno CS Sex-determining region Y box 4 is a transforming oncogene in human prostate cancer cells Cancer Res 2006 66 4011 4019 10.1158/0008-5472.CAN-05-3055 16618720

[B31] Luo JH Yu YP Cieply K Lin F Deflavia P Dhir R Finkelstein S Michalopoulos G Becich M Gene expression analysis of prostate cancers Mol Carcinog 2002 33 25 35 10.1002/mc.10018 11807955

[B32] Magee JA Araki T Patil S Ehrig T True L Humphrey PA Catalona WJ Watson MA Milbrandt J Expression profiling reveals hepsin overexpression in prostate cancer Cancer Res 2001 61 5692 5696 11479199

[B33] Singh D Febbo PG Ross K Jackson DG Manola J Ladd C Tamayo P Renshaw AA D'Amico AV Richie JP Lander ES Loda M Kantoff PW Golub TR Sellers WR Gene expression correlates of clinical prostate cancer behavior Cancer Cell 2002 1 203 209 10.1016/S1535-6108(02)00030-2 12086878

[B34] Taylor BS Schultz N Hieronymus H Gopalan A Xiao Y Carver BS Arora VK Kaushik P Cerami E Reva B Antipin Y Mitsiades N Landers T Dolgalev I Major JE Wilson M Socci ND Lash AE Heguy A Eastham JA Scher HI Reuter VE Scardino PT Sander C Sawyers CL Gerald WL Integrative genomic profiling of human prostate cancer Cancer Cell 2010 18 11 22 10.1016/j.ccr.2010.05.026 20579941 PMC3198787

[B35] Tomlins SA Mehra R Rhodes DR Cao X Wang L Dhanasekaran SM Kalyana-Sundaram S Wei JT Rubin MA Pienta KJ Shah RB Chinnaiyan AM Integrative molecular concept modeling of prostate cancer progression Nat Genet 2007 39 41 51 10.1038/ng1935 17173048

[B36] Vanaja DK Cheville JC Iturria SJ Young CY Transcriptional silencing of zinc finger protein 185 identified by expression profiling is associated with prostate cancer progression Cancer Res 2003 63 3877 3882 12873976

[B37] Varambally S Yu J Laxman B Rhodes DR Mehra R Tomlins SA Shah RB Chandran U Monzon FA Becich MJ Wei JT Pienta KJ Ghosh D Rubin MA Chinnaiyan AM Integrative genomic and proteomic analysis of prostate cancer reveals signatures of metastatic progression Cancer Cell 2005 8 393 406 10.1016/j.ccr.2005.10.001 16286247

[B38] Wallace TA Prueitt RL Yi M Howe TM Gillespie JW Yfantis HG Stephens RM Caporaso NE Loffredo CA Ambs S Tumor immunobiological differences in prostate cancer between African-American and European-American men Cancer Res 2008 68 927 936 10.1158/0008-5472.CAN-07-2608 18245496

[B39] Welsh JB Sapinoso LM Su AI Kern SG Wang-Rodriguez J Moskaluk CA Frierson HF Jr Hampton GM Analysis of gene expression identifies candidate markers and pharmacological targets in prostate cancer Cancer Res 2001 61 5974 5978 11507037

[B40] Lin B White JT Lu W Xie T Utleg AG Yan X Yi EC Shannon P Khrebtukova I Lange PH Goodlett DR Zhou D Vasicek TJ Hood L Evidence for the presence of disease-perturbed networks in prostate cancer cells by genomic and proteomic analyses: a systems approach to disease Cancer Res 2005 65 3081 3091 15833837 10.1158/0008-5472.CAN-04-3218

[B41] Maher CA Kumar-Sinha C Cao X Kalyana-Sundaram S Han B Jing X Sam L Barrette T Palanisamy N Chinnaiyan AM Transcriptome sequencing to detect gene fusions in cancer Nature 2009 458 97 101 10.1038/nature07638 19136943 PMC2725402

[B42] Maher CA Palanisamy N Brenner JC Cao X Kalyana-Sundaram S Luo S Khrebtukova I Barrette TR Grasso C Yu J Lonigro RJ Schroth G Kumar-Sinha C Chinnaiyan AM Chimeric transcript discovery by paired-end transcriptome sequencing Proc Natl Acad Sci U S A 2009 106 12353 12358 10.1073/pnas.0904720106 19592507 PMC2708976

[B43] Lapointe J Li C Giacomini CP Salari K Huang S Wang P Ferrari M Hernandez-Boussard T Brooks JD Pollack JR Genomic profiling reveals alternative genetic pathways of prostate tumorigenesis Cancer Res 2007 67 8504 8510 10.1158/0008-5472.CAN-07-0673 17875689

[B44] Paris PL Andaya A Fridlyand J Jain AN Weinberg V Kowbel D Brebner JH Simko J Watson JE Volik S Albertson DG Pinkel D Alers JC van der Kwast TH Vissers KJ Schroder FH Wildhagen MF Febbo PG Chinnaiyan AM Pienta KJ Carroll PR Rubin MA Collins C van Dekken H Whole genome scanning identifies genotypes associated with recurrence and metastasis in prostate tumors Hum Mol Genet 2004 13 1303 1313 10.1093/hmg/ddh155 15138198

[B45] Khan AP Poisson LM Bhat VB Fermin D Zhao R Kalyana-Sundaram S Michailidis G Nesvizhskii AI Omenn GS Chinnaiyan AM Sreekumar A Quantitative proteomic profiling of prostate cancer reveals a role for miR-128 in prostate cancer Mol Cell Proteomics 2010 9 298 312 10.1074/mcp.M900159-MCP200 19955085 PMC2830841

[B46] Tomlins SA Rubin MA Chinnaiyan AM Integrative biology of prostate cancer progression Annu Rev Pathol 2006 1 243 271 10.1146/annurev.pathol.1.110304.100047 18039115

[B47] Vellaichamy A Sreekumar A Strahler JR Rajendiran T Yu J Varambally S Li Y Omenn GS Chinnaiyan AM Nesvizhskii AI Proteomic interrogation of androgen action in prostate cancer cells reveals roles of aminoacyl tRNA synthetases PLoS One 2009 4 e7075 10.1371/journal.pone.0007075 19763266 PMC2740864

[B48] Haffner MC Aryee MJ Toubaji A Esopi DM Albadine R Gurel B Isaacs WB Bova GS Liu W Xu J Meeker AK Netto G De Marzo AM Nelson WG Yegnasubramanian S Androgen-induced TOP2B-mediated double-strand breaks and prostate cancer gene rearrangements Nat Genet 2010 42 668 675 10.1038/ng.613 20601956 PMC3157086

[B49] Mani RS Tomlins SA Callahan K Ghosh A Nyati MK Varambally S Palanisamy N Chinnaiyan AM Induced chromosomal proximity and gene fusions in prostate cancer Science 2009 326 1230 10.1126/science.1178124 19933109 PMC2935583

[B50] Armisen R Marcelain K Simon F Tapia JC Toro J Quest AF Stutzin A TRPM4 enhances cell proliferation through up-regulation of the beta-catenin signaling pathway J Cell Physiol 2011 226 103 109 10.1002/jcp.22310 20625999

[B51] Fraser SP Pardo LA Ion channels: functional expression and therapeutic potential in cancer. Colloquium on Ion Channels and Cancer EMBO Rep 2008 9 512 515 10.1038/embor.2008.75 18451877 PMC2427390

[B52] Prevarskaya N Flourakis M Bidaux G Thebault S Skryma R Differential role of TRP channels in prostate cancer Biochem Soc Trans 2007 35 133 135 10.1042/BST0350133 17233619

[B53] Roginski RS Mohan Raj BK Birditt B Rowen L The human GRINL1A gene defines a complex transcription unit, an unusual form of gene organization in eukaryotes Genomics 2004 84 265 276 10.1016/j.ygeno.2004.04.004 15233991

[B54] Le TP Sun M Luo X Kraus WL Greene GL Mapping ERbeta genomic binding sites reveals unique genomic features and identifies EBF1 as an ERbeta interactor PLoS One 2013 8 e71355 10.1371/journal.pone.0071355 23951143 PMC3738513

[B55] Hartman J Strom A Gustafsson JA Current concepts and significance of estrogen receptor beta in prostate cancer Steroids 2012 77 1262 1266 10.1016/j.steroids.2012.07.002 22824289

[B56] Aung CS Hill MM Bastiani M Parton RG Parat MO PTRF-cavin-1 expression decreases the migration of PC3 prostate cancer cells: role of matrix metalloprotease 9 Eur J Cell Biol 2011 90 136 142 10.1016/j.ejcb.2010.06.004 20732728

[B57] Gould ML Williams G Nicholson HD Changes in caveolae, caveolin, and polymerase 1 and transcript release factor (PTRF) expression in prostate cancer progression Prostate 2010 70 1609 1621 10.1002/pros.21195 20564315

[B58] Nassar ZD Moon H Duong T Neo L Hill MM Francois M Patron RG Parat MO PTRF/Cavin-1 decreases prostate cancer angiogenesis and lymphangiogenesis Oncotarget 2013 4 1844 1855 24123650 10.18632/oncotarget.1300 PMC3858569

[B59] Moon H Lee CS Inder KL Sharma S Choi E Black DM Le Cao KA Winterford C Coward JI Ling MT Craik DJ Parton RG Russell PJ Hill MM the Australian Prostate Cancer BioResource PTRF/cavin-1 neutralizes non-caveolar caveolin-1 microdomains in prostate cancer Oncogene 2013 doi:10.1038/onc.2013.315 10.1038/onc.2013.315 23934189

[B60] Buchet-Poyau K Courchet J Le Hir H Seraphin B Scoazec JY Duret L Domon-Dell C Freund JN Billaud M Identification and characterization of human Mex-3 proteins, a novel family of evolutionarily conserved RNA-binding proteins differentially localized to processing bodies Nucleic Acids Res 2007 35 1289 1300 10.1093/nar/gkm016 17267406 PMC1851655

[B61] Mougeot JL Bahrani-Mougeot FK Lockhart PB Brennan MT Microarray analyses of oral punch biopsies from acute myeloid leukemia (AML) patients treated with chemotherapy Oral Surg Oral Med Oral Pathol Oral Radiol Endod 2011 112 446 452 10.1016/j.tripleo.2011.05.009 21862359

[B62] Wang ZG Ackerman SH The assembly factor Atp11p binds to the beta-subunit of the mitochondrial F(1)-ATPase J Biol Chem 2000 275 5767 5772 10.1074/jbc.275.8.5767 10681564

[B63] Sanchez-Cenizo L Formentini L Aldea M Ortega AD Garcia-Huerta P Sanchez-Arago M Cuezva JM Up-regulation of the ATPase inhibitory factor 1 (IF1) of the mitochondrial H + -ATP synthase in human tumors mediates the metabolic shift of cancer cells to a Warburg phenotype J Biol Chem 2010 285 25308 25313 10.1074/jbc.M110.146480 20538613 PMC2919093

[B64] Trobridge GD Wu RA Hansen M Ironside C Watts KL Olsen P Beard BC Kiem HP Cocal-pseudotyped lentiviral vectors resist inactivation by human serum and efficiently transduce primate hematopoietic repopulating cells Mol Ther 2010 18 725 733 10.1038/mt.2009.282 19997089 PMC2862537

[B65] Kent WJ BLAT–the BLAST-like alignment tool Genome Res 2002 12 656 664 10.1101/gr.229202 11932250 PMC187518

[B66] Chapman DG The Estimation of Biological Populations Ann Math Stat 1954 25 1 14 10.1214/aoms/1177728844

[B67] Krebs CJ From Chapter 2 Estimating Abundance: Mark-Recapture Techniques Ecological Methodology 1998 2 Menlo Park, California: Addison Wesley Longman, Inc. 35 41

