# Supplementary material for: A novel approach to identify driver genes involved in androgen-independent prostate cancer
Source: Mol Cancer. 2014 May 23;13:120. doi: 10.1186/1476-4598-13-120 (PMC4098713; doi:10.1186/1476-4598-13-120)
Supplement: Additional file 1: Figure S1 — Determining the clonality of LV integrants. [file 1476-4598-13-120-S1.pdf]

# Supplemental Figure 1. Schinke et al.

**Schnabel Equation:**

$$N = \frac{\Sigma (n_i M_i)}{(\Sigma m_i) + 1}$$

| SVM A | | | | |
|---|---|---|---|---|
| **Sample (i)** | $n_i$ | $m_i$ | $M_i$ | $n_iM_i$ |
| **1** | 32 | 0 | 0 | 0 |
| **2** | 36 | 0 | 32 | 1152 |
| **3** | 43 | 1 | 68 | 2924 |
| **4** | 51 | 0 | 111 | 5661 |
| **5** | 29 | 0 | 162 | 4698 |
| | | | N | $7.22 \times 10^3$ |

**Supplemental Figure S1. Determining the clonality of LV integrants.** To estimate the clonality of the transduced LNcaP cells the Schnabel method of multiple census Mark-Recapture was used. As an example, the data is shown for the clonality calculation of sample SVM A. $n_i$ – the number of unique integration sites captured in the *i*th sample; $m_i$ – the number of previously identified integration sites captured in the *i*the sample; $M_i$ – the number of previously identified integration sites in the population prior to the *i*th sample, where $M_1 = 0$; N – the unknown size of the population just prior to the first sample. Each new sequencing sample is considered the next subsequent *i*th sample. If an integration site was recovered, or "recaptured", in a previous *i*th sample, this integrant is counted under the variable $m_i$. The variable $\Sigma\ m_i$ is inversely proportional to the clonality of the population, as this variable represents the amount of recapture present in the analysis of the population. The less recapture there is, the more polyclonal the population is. The variable $M_i$ comprises all of the integrations previously identified prior to the current survey ($M_i = n_{i-1} + n_{i-2}$ + ..., etc.). For example, $M_i$ when $i = 2$ is equivalent to 32, or $n_1$. Similarly, when $i = 3$, $M_i$ equals 68 or $n_1 + n_2$. Thus, the cumulative nature of $M_i$ is of crucial importance.
