# Supplementary material for: A novel approach to identify driver genes involved in androgen-independent prostate cancer
Source: Mol Cancer. 2014 May 23;13:120. doi: 10.1186/1476-4598-13-120 (PMC4098713; doi:10.1186/1476-4598-13-120)
Supplement: Additional file 4: Figure S2 — Oncomine meta-analysis of expression in prostate cancer tissue of genes near vector proviruses. [file 1476-4598-13-120-S4.pdf]

Supplemental Figure 2. Oncomine meta-analysis of expression in prostate cancer tissue of genes near vector proviruses

1A. Expression analysis across 16 datasets

1A-1. overexpressed genes

1. TRPM4

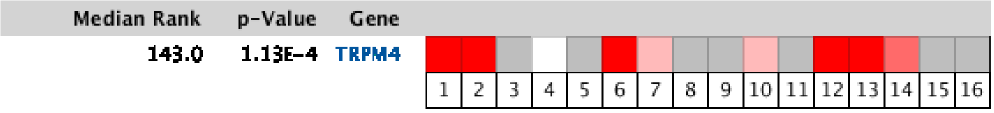

2. MEX3D

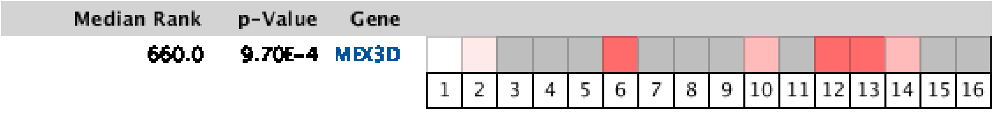

3. ATPAF1

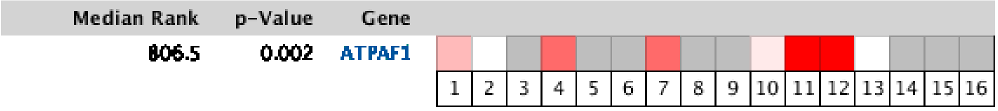

1A-2. underexpressed genes

4. PTRF

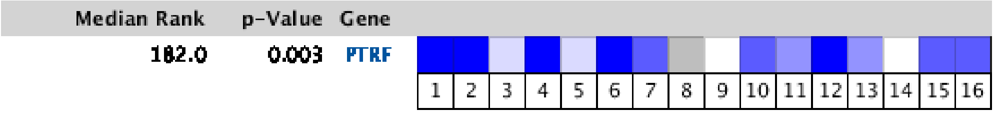

5. GCOM1

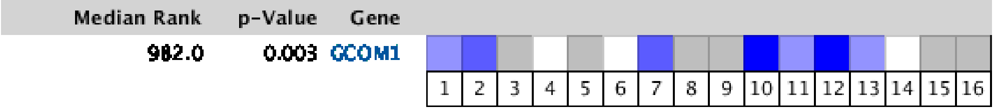

Legend

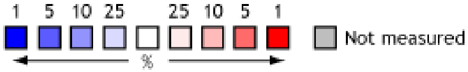

The rank for a gene is the median rank for that gene across each of the analyses. The p-Value for a gene is its p-Value for the median-ranked analysis.

Datasets:
1. Prostate Carcinoma vs. Normal *Arredouani, Clin Cancer Res, 2009*
2. Prostate Carcinoma vs. Normal *Grasso, Nature, 2012*
3. Prostate Carcinoma vs. Normal *Holzbeierlein, Am J Pathol, 2004*
4. Prostate Carcinoma vs. Normal *Lapointe, Proc Natl Acad Sci U S A, 2004*
5. Prostate Carcinoma vs. Normal *LaTulippe, Cancer Res, 2002*
6. Prostate Carcinoma vs. Normal *Liu, Cancer Res, 2006*
7. Prostate Carcinoma vs. Normal *Luo, Mol Carcinog, 2002*
8. Prostate Carcinoma vs. Normal *Magee, Cancer Res, 2001*
9. Prostate Carcinoma vs. Normal *Singh, Cancer Cell, 2002*
10. Prostate Carcinoma vs. Normal *Taylor, Cancer Cell, 2010*
11. Prostate Carcinoma Epithelia vs. Normal *Tomlins, Nat Genet, 2007*
12. Prostate Adenocarcinoma vs. Normal *Vanaja, Cancer Res, 2003*
13. Prostate Carcinoma vs. Normal *Varambally, Cancer Cell, 2005*
14. Prostate Adenocarcinoma vs. Normal *Wallace, Cancer Res, 2008*
15. Prostate Carcinoma vs. Normal *Welsh, Cancer Res, 2001*
16. Prostate Carcinoma vs. Normal *Yu, J Clin Oncol, 2004*

Supplemental Figure 2. Continued

1B. Analysis in individual datasets

1B-1. overexpressed genes

1. TRPM4 is over-expressed (p<0.005) in prostate cancer tissue in 7 datasets:

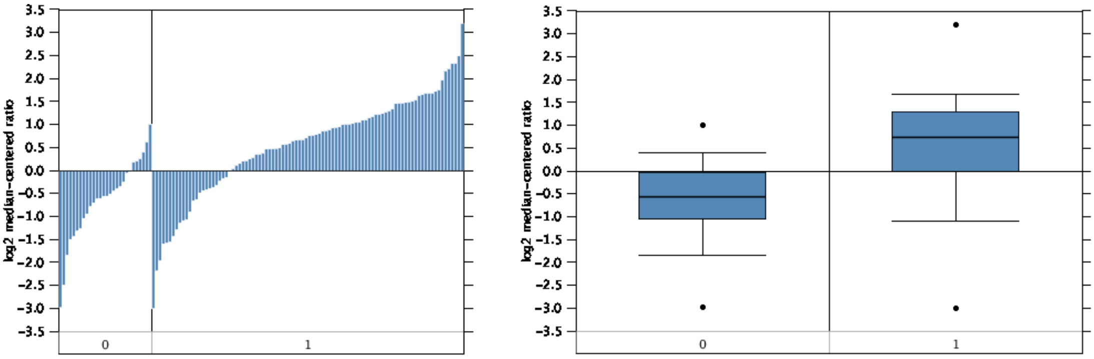

Legend
0. No value (28), 1. Prostate Carcinoma (94)
Grasso, *Nature, 2012,*
mRNA 19,189 measured genes, Agilent Human Genome 44K, Reporter ID: A_23_P153529
Over expression Gene Rank: 91 (in top 1%) P value: 7.08E-11

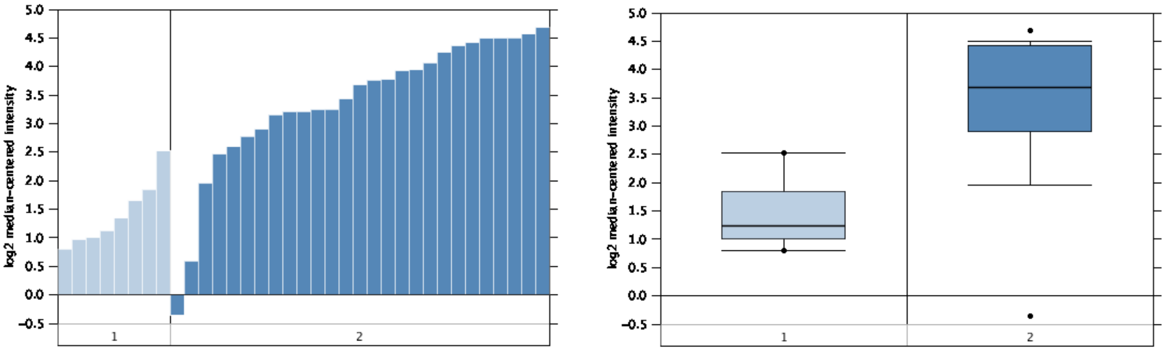

Legend
1. Prostate Gland (8 samples) 2. Prostate Adenocarcinoma (27 samples)
Vanaja *Cancer Res,* 2003
mRNA 17,779 measured genes, Human Genome U133A, U133B Array Reporter ID: A_23_P153529
Over-expression Gene Rank: 86 (in top 1%) P-value: 4.24E-7

- 5 additional datasets with TRPM4 over-expression in prostate cancer tissue, p<0.005:

Liu*, Cancer Res,* 2006*,* 57 samples, Human Genome U133A Array, P-value: 2.57E-6
Varambally *Cancer Cell*, 2005, 19 samples, Human Genome U133 Plus 2.0 Array, P-value: 3.94E-6
Arredouani, *Clin Cancer Res,* 2009, 21 samples, Human Genome U133 Plus 2.0 Array, P-value: 1.13E-4
Wallace, *Cancer Res*, 2008, 89 samples, Human Genome U133A 2.0 Array, P-value: 1.59E-4
Taylor, Cancer Cell, 2010, 185 samples, Platform not pre-defined in Oncomine, P-value: 5.59E-4

Supplemental Figure 2. Continued

3. ATPAF1 is over-expressed (p<0.005) in prostate cancer tissue in 5 datasets:

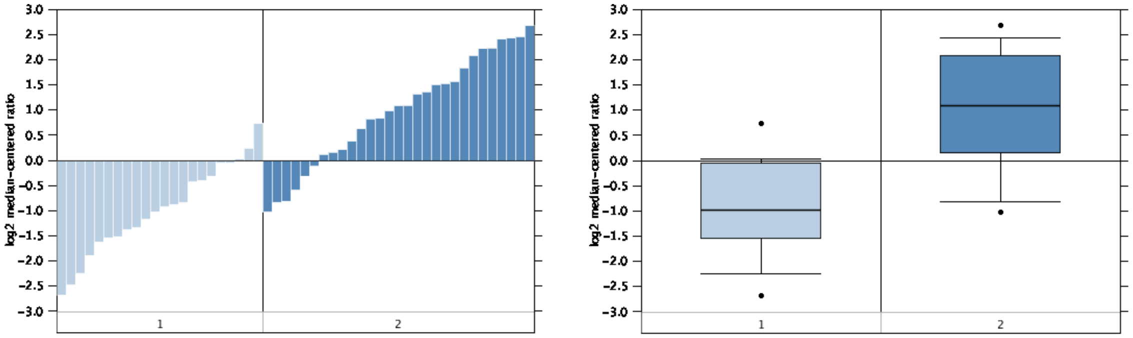

Legend
1. Prostate Gland (22 samples), 2. Prostate Carcinoma (29 samples)
Tomlins, *Nat Genet,* 2007
mRNA 10,656 measured genes, Platform not pre-defined in Oncomine, Reporter ID: IMAGE:85384
Over-expression Gene Rank: 16 (in top 1%) P-value: 3.48E-9

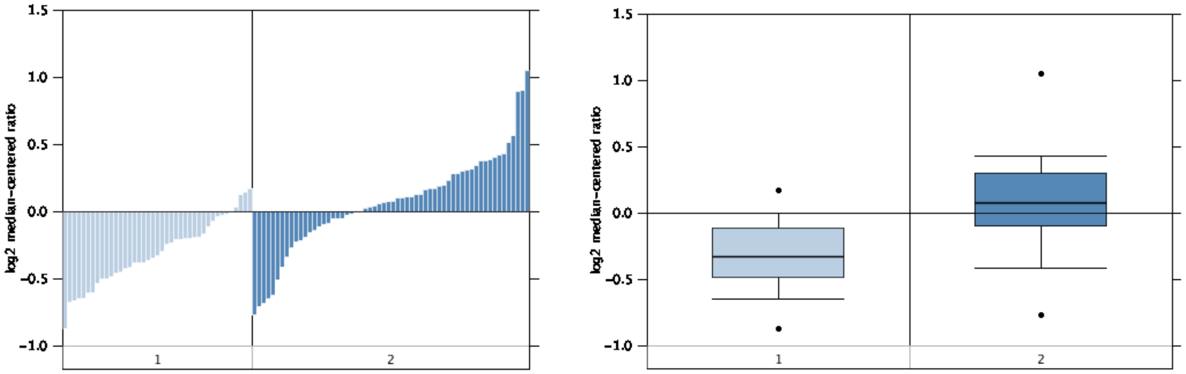

Legend
1. Prostate Gland (41 samples), 2. Prostate Carcinoma (60 samples)
Lapointe *Proc Natl Acad Sci U S A* 2004,
mRNA 10,166 measured genes, Platform not pre-defined in Oncomine Reporter ID: IMAGE:85384
Over-expression Gene Rank: 281 (in top 3%) P-value: 1.25E-8

- 3 additional datasets with ATPAF1 over-expression in prostate cancer tissue, p<0.005:

Vanaja, Cancer Res, 2003, Human Genome U133B Array, P-value: 1.23E-6
Luo, *Mol Carcinog*, 2002, Hu35KsubB Array, P-value: 0.002
Arredouani, *Clin Cancer Res,* 2009, Human Genome U133 Plus 2.0 Array, P-value: 0.004

Supplemental Figure 2. Continued

<u>1B-2. Under-expressed genes</u>

4. PTRF is under-expressed (p<0.005) in prostate cancer tissue in 10 datasets:

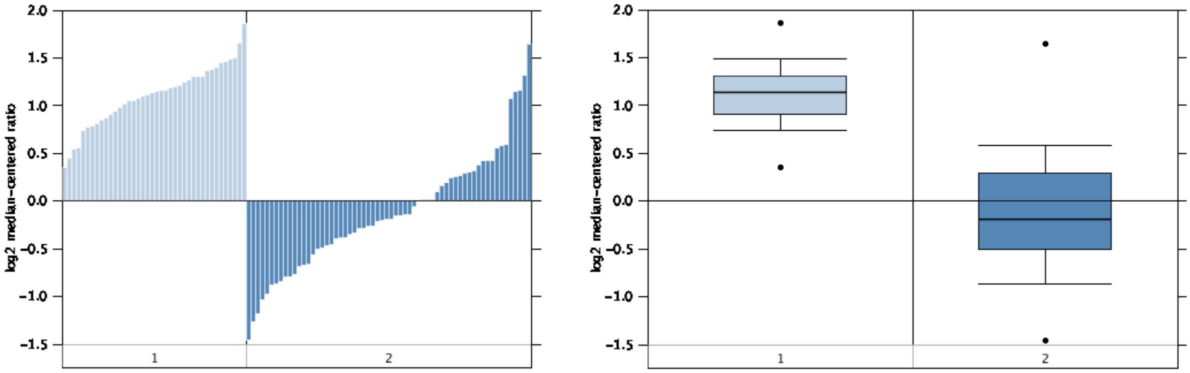

Legend
1. Prostate Gland (40 samples), 2. Prostate Carcinoma (62 samples)
Lapointe, Proc Natl Acad Sci U S A, 2004
mRNA 10,166 measured genes, Platform not pre-defined in Oncomine, Reporter ID: IMAGE:809473
Under-expression Gene Rank: 8 (in top 1%) P-value: 6.09E-23

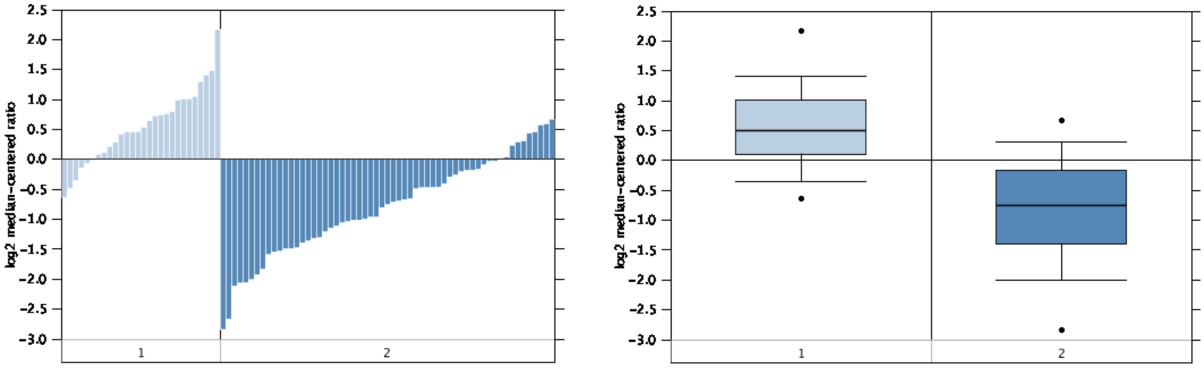

Legend
1. Prostate Gland (28 samples), 2. Prostate Carcinoma (59 samples)
Grasso, *Nature* 2012
mRNA 19,189 measured genes, Agilent Human Genome 44K, Reporter ID: A_23_P394064
Under-expression Gene Rank: 75 (in top 1%) P-value: 2.40E-12

- 8 additional datasets with PTRF under-expression in prostate cancer tissue, p<0.005:

Arredouani, *Clin Cancer Res,* 2009, Human Genome U133 Plus 2.0 Array, P-value: 2.80E-7
Welsh, *Cancer Res,* 2001, Human Genome U95A-Av2 Array, P-value: 8.11E-6
Liu, *Cancer Res,* 2006, Human Genome U133A Array, P-value: 4.40E-8
Taylor, *Cancer Cell*, 2010, Platform not pre-defined in Oncomine, P-value: 9.55E-8
Yu, *J Clin Oncol* 2004, Human Genome U95A-Av2 Array, P-value: 3.19E-6
Vanaja, *Cancer Res*, 2003, Human Genome U133B Array, P-value: 5.28E-6

Supplemental Figure 2. Continued

1B-2. Under-expressed genes

4. PTRF is under-expressed (p<0.005) in prostate cancer tissue in 10 datasets:

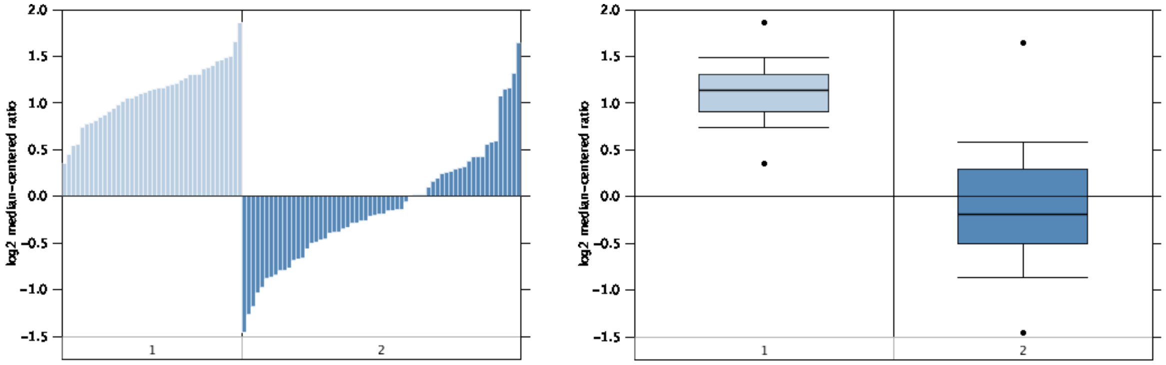

Legend
1. Prostate Gland (40 samples), 2. Prostate Carcinoma (62 samples)
Lapointe, Proc Natl Acad Sci U S A, 2004
mRNA 10,166 measured genes, Platform not pre-defined in Oncomine, Reporter ID: IMAGE:809473
Under-expression Gene Rank: 8 (in top 1%) P-value: 6.09E-23

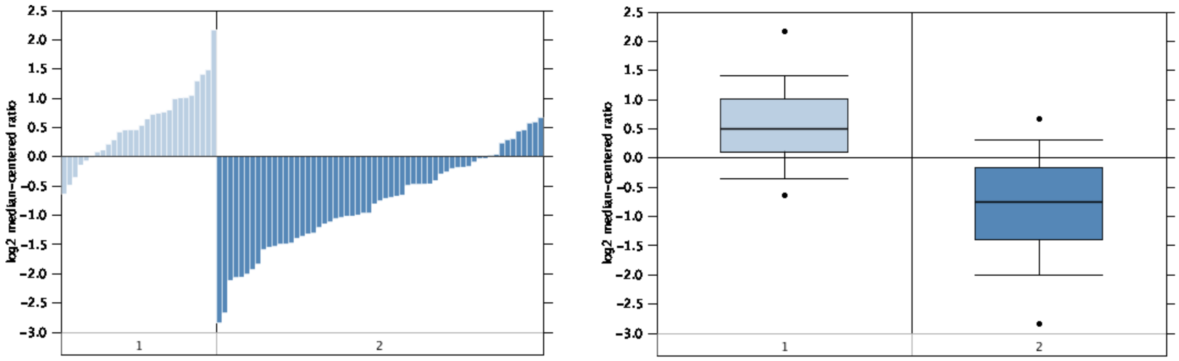

Legend
1. Prostate Gland (28 samples), 2. Prostate Carcinoma (59 samples)
Grasso, *Nature* 2012
mRNA 19,189 measured genes, Agilent Human Genome 44K, Reporter ID: A_23_P394064
Under-expression Gene Rank: 75 (in top 1%) P-value: 2.40E-12

- 8 additional datasets with PTRF under-expression in prostate cancer tissue, p<0.005:

Arredouani, *Clin Cancer Res,* 2009, Human Genome U133 Plus 2.0 Array, P-value: 2.80E-7
Welsh, *Cancer Res,* 2001, Human Genome U95A-Av2 Array, P-value: 8.11E-6
Liu*, Cancer Res,* 2006, Human Genome U133A Array, P-value: 4.40E-8
Taylor, *Cancer Cell*, 2010, Platform not pre-defined in Oncomine, P-value: 9.55E-8
Yu, *J Clin Oncol* 2004, Human Genome U95A-Av2 Array, P-value: 3.19E-6
Vanaja, *Cancer Res*, 2003, Human Genome U133B Array, P-value: 5.28E-6
Luo, *Mol Carcinog*, 2002, Hu35KsubB Array, P-value: 0.003
Tomlins, *Nat Genet,* 2007, Platform not pre-defined in Oncomine, P-value: 0.004

Supplemental Figures Schinke et al.

Supplemental Figure 2. Continued

5. GCOM1 is underexpressed (p<0.005) in prostate cancer tissue in 6 datasets:

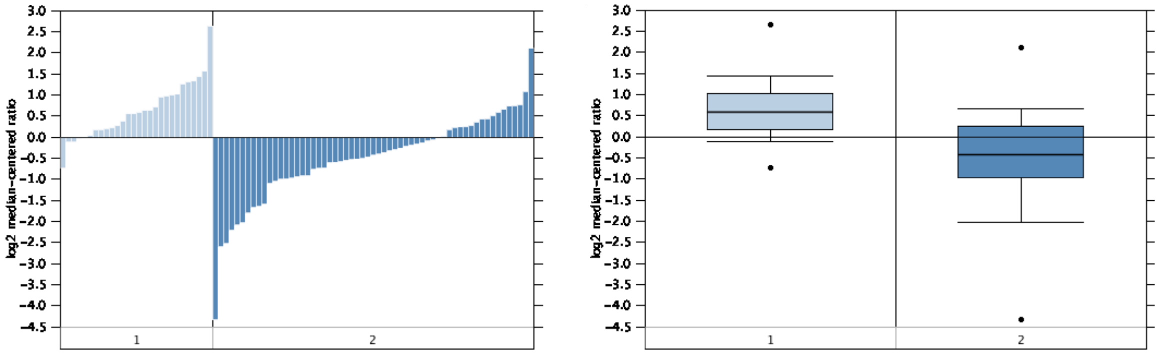

Legend
1. Prostate Gland (28 samples), 2. Prostate Carcinoma (59 samples)

Grasso, Nature, 2012
mRNA 19,189 measured genes, Agilent Human Genome 44K
Under-expression Gene Rank: 465 (in top 3%) P-value: 2.00E-8

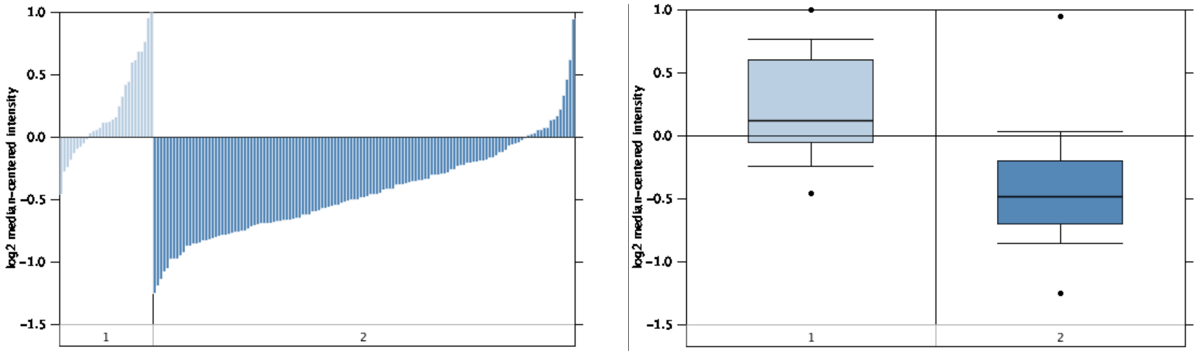

Legend
1. Prostate Gland (29 samples), 2. Prostate Carcinoma (131 samples)
Taylor Prostate, *Cancer Cell* 2010
mRNA 22,238 measured genes, Platform not pre-defined in Oncomine
Under-expression Gene Rank: 199 (in top 1%) P-value: 6.67E-11

- 4 additional datasets with GCOM1 under-expression in prostate cancer tissue, p<0.005:

Vanaja, *Cancer Res*, 2003, Human Genome U133B Array, P-value: 1.27E-8
Arredouani, *Clin Cancer Res,* 2009, Human Genome U133 Plus 2.0 Array, -value: 0.003
Tomlins, *Nat Genet,* 2007, Platform not pre-defined in Oncomine, P-value: 0.003
Varambally Cancer Cell, 2005, 19 samples, Human Genome U133 Plus 2.0 Array, P-value: 0.004
