# Supplementary material for: A novel approach to identify driver genes involved in androgen-independent prostate cancer
Source: Mol Cancer. 2014 May 23;13:120. doi: 10.1186/1476-4598-13-120 (PMC4098713; doi:10.1186/1476-4598-13-120)
Supplement: Additional file 5: Figure S3 — Localization of vector proviruses to the human genome. [file 1476-4598-13-120-S5.pdf]

Supplemental Figure S3: Localization of vector proviruses to the human genome.

Sequences obtained by shuttle vector rescue are localized to the human genome using BLAT to identify the integration site and nearby genes.

1) ATPAF1

LTR-chromosome junction sequence read after shuttle vector rescue

```
AGTGTGGAAAATCTCTAGCACCTCCTACCTCAGCCTCCTGAGTAGCTGGGACTACAGGCTCACACCAATGC
CCCCAACCATTTTGTCATAGTCTACTTGTTAGAAGGAAGTCACTGAGTATAGCCATTACCCAAGGAAAGGG
AAATTAGGCTCCATCAAAGGGAGGAATATCTTAAAATTTGTGGGCAAATTTTAAAACCATCACAGCTGGCT
CTGGGAGTACTTTTCTGACCAAGGTACTATCATAACAGGTTTTATTCAAGTGTATATCTGGTTAGTCAAGA
ACTTAGTTCTCACTCACAAACTGGTTCTTAGAGCTGGGTAACTATTGCTGTCTTATTCCCCCCTTGAAATA
```

Query sequence localized to chromosome 1 using BLAT

```
ggcttgatag cagaactcat tctccatcat ttttcatttt ttcatagtct  47102416
tttttttttt ttaaggggct cactacatta cccagacagg ctcaagcaat  47102366
CCTCCTACCT CAGCCTCCTG AGTAGCTGGG ACTACAGGCT CACACCAATG  47102316
CCCCCAACCA TTTTGTCATA GTCTACTTGT TAGAAGGAAG TCACTGAGTA  47102266
TAGCCATTAC CCAAGGAAAG GGAAATTAGG CTCCATCAAA GGGAGGAATA  47102216
TCTTAAAATT TGTGGGCAAA TTTTAAAACC ATCACAGCTG GCTCTGGGAG  47102166
```

2) TRPM4

LTR-chromosome junction sequence read after shuttle vector rescue

```
AGTGTGGAAAATCTCTAGCAGCCACCACACCTGGCTAATTTTGTAGTTTTAGTAGAGATGGGGTTTCTCCA
TATTGGTCAGGCTGGTCTCGAACTCCCGACCTCGGGTGATCCGCCCGCCTCGGCCTCCCAAAGTGCTGGGA
TTACAGGCATGAGCCACTGTGCCCAGCCTTTTTTGTATTTTTAGTAGAGATGGGGTTTCACTATGTTGCCC
AGGCTGGTCTCGAACTCCTGGGCTAAAGCTATCTGCCCATCTCGGCCTCCCAACGTGCTGGGATTACAGGC
GTGAGCCATTGTGCTTAGGCCAGAAGAATTTTACGGGGGAAATACACACTAAGATAGAAACTCCCCAGATA
```

Query sequence localized to chromosome 19 using BLAT

```
gagtgcaata gtgccatctc agctcactgc aacctccgcc tcccgggctc  49678431
aagtgattct cctgcctcag cctcccaagt agctgggatt acaggcatgc  49678481
GCCACCACAC CTGGCTAATT TTGTAGTTTT AGTAGAGATG GGGTTTCTCC  49678531
ATATTGGTCA GGCTGGTCTC GAACTCCCGA CCTCGGGTGA TCCGCCCGCC  49678581
TCGGCCTCCC AAAGTGCTGG GATTACAGGC ATGAGCCACT GTGCCCAGCC  49678631
TTTTTTGTAT TTTTAGTAGA GATGGGGTTT CACTATGTTG CCCAGGCTGG  49678681
```

3) GCOM1

LTR-chromosome junction sequence read after shuttle vector rescue

```
AGTGTGGAAAATCTCTAGCACATATGAAGAGTCAGGAAAATAGTACCCACTCCTAAGGGAAAAGAGAATCA
ACAGACACCAATCCTGATGTAACTCTGATGTTGGAATTTTCAGACAAAGACTTTAATGCAGCCATCATAAC
AATGATGAGTCAGACAAAGAAAAATATGCTTACAATTAATGAAGTAGTAAAAAAAATAGAAAATACAAAAA
AGAACTAAATGAAAATTTTAAGAACAGGAAAGTACAATACCTGAATTTTTTTTTAAAAGTCACTAGATGGA
CTTTAAGACTGAAGAATGGTAAAGGAAAAAAGTCAGTAAAATTTTACAATACAATTTAAATATTAAATTAT
```

Query sequence localized to human genome chromosome 15 using BLAT

caggcaggat gtagttgggt ctggctaaat tcattgcctt ttaaaacaag 57964177
aacccaacaa agcactcaca atgtccagct tataatccaa aattatgtga 57964127
CATATaAAGA GTCgGGAAAA TAGTACCCAC TCCTAAGGGA AAAGAGAATC 57964077
AACAGACACC AATCCTGATG TAACTaTGAT GTTGGAATTT TCAGACAAAG 57964027
ACTTTAATGC AGCCATCATA ACAATGATcA GTCAGACAAA GAAAAATATG 57963977
CTTACAATTA ATGAAGTAGT AAAAAAAATA GAAAATACAA AAAAGAACTA 57963927

4) PTRF

LTR-chromosome junction sequence read after shuttle vector rescue

**AGTGTGGAAAATCTCTAGCAGGTTGTTTTAGTGTTCCATTTCAATTTTGATACACAGAATTAGAATAGCAT
CCAGATGTGGGTCTGTTACAGCTAGACTACTAGATCCTTCAAAATCCAAGTACTAGTATGTCTATTAAAAT
ACCATAAGATCACATTGGCTAGTTACAATGGTTGGTTTGTGGGTTACTTAAAAATCAACTAAAATTCTTTT
TTTTTTTTGAGATGGAGTTTTGCTCTTGTTGCCTAGGCTGGAATGCAATGACACAATCTTGGCTCACTGCC
ACCTCTGCCTCCCAGGTTCAAGCAATTCCCCTGCCTTAGCCTCCTGAGTAGCTGGGATTACAGGCATGTGC**

Query sequence localized to human genome chromosome 17 using BLAT

ctctcctaac ctcactcctg ttgcctgcat cttcttgctg agcaaaatat 40512915
tcaaggtctt caactcctca caccctggtt gtccctccct ggatgtgttt 40512865
GGTTGTTTTA GTGTTCCATT TCAATTTTGA TACACAGAAT TAGAATAGCA 40512815
TCCAGATGTG GGTCTGTTAC AGCTAGACTA CTAGATCCTT CAAAATCCAA 40512765
GTACTAGTAT GTCTATTAAA ATACCATAAG ATCACATTGG CTAGTTACAA 40512715
TGGTTGGTTT GTGGGTTACT TAAAAATCAA CTAAAATTCt TTTTTTTTTT 40512665

5) MEX3D

**AGTGTGGAAAATCTCTAGCAGATTCCCCTTGTGAGCCTCCATGTCTGAAGCCAGTCTTTTGTCACTTGACC
TCGGCAGTGATGCCCCATTGCTCATGCTGCGTTCTACTCATTAGAAGCCTGTGCCCGGCCGGGCGCGGTGG
CTCACGCCTGTAATCCCAGCACTTTGGGAGGCCGAGGCGGGCGGATCACGAGGTCAGGAGATTGAGACCAT
CCTGGCTAACACGGTGAAACCCCATCTCTACTAAAAATGCAAAAAATTAGCCAGGCGCAGTGACGGGCGCC
TGTAGTCCCAGCTACTCGGGAGGCTGAGGCAGGAGAATTGCTTGAACCCGGGAGGTGGAGATTGCAGTGAG**

Query sequence localized to human genome chromosome 19 using BLAT

cttaattttg tgtagagatg gggtctcact ctgttgtcta ggctggtctc 1600669
aaactcctag gctcaagtga gcctcctgcc tcaacctccc aaagtgctgg 1600619
GATTCCCCTT GTGAGCCTCC ATGTCTGAAG CCAGTCTTTT GTCACTTGAC 1600569
CTCGGCAGTG ATGCCCCATT GCTCATGCTG CGTTCTACTC ATTAGAAGCC 1600519
TGTGCCCGGC CGGGCGCGGT GGCTCACGCC TGTAATCCCA GCACTTTGGG 1600469
AGGCCGAGGC GGGCGGATCA CGAGGTCAGG AGATTGAGAC CATCCTGGCT 1600419
