# Supplementary material for: A novel approach to identify driver genes involved in androgen-independent prostate cancer
Source: Mol Cancer. 2014 May 23;13:120. doi: 10.1186/1476-4598-13-120 (PMC4098713; doi:10.1186/1476-4598-13-120)
Supplement: Additional file 6: Figure S4 — Proviral integration sites displayed using the UCSC genome browser. The positions of the proviral integration site is indicated by the vertical line on images generated by the University of California, Santa Cruz genome browser. [file 1476-4598-13-120-S6.pdf]

Supplemental Figure S4. Proviral integration sites displayed using the UCSC genome browser. The positions of the proviral integration site is indicated by the vertical line on images generated by the University of California, Santa Cruz genome browser.

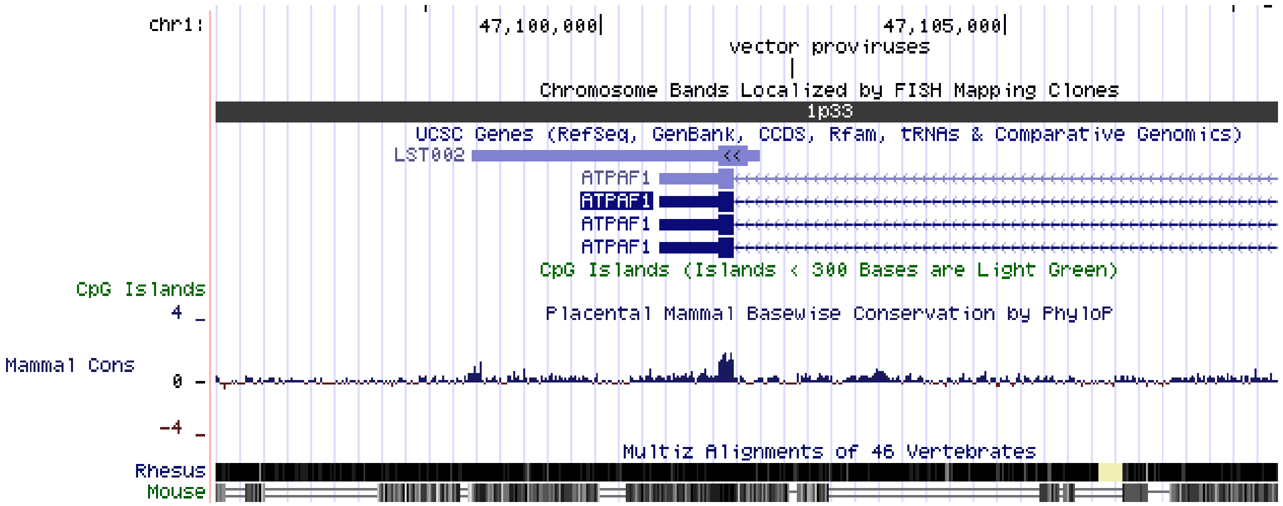

S4A. Proviral integration site in ATP synthase mitochondrial F1 complex assembly factor 1 (*ATPAF1*) on chromosome 1.

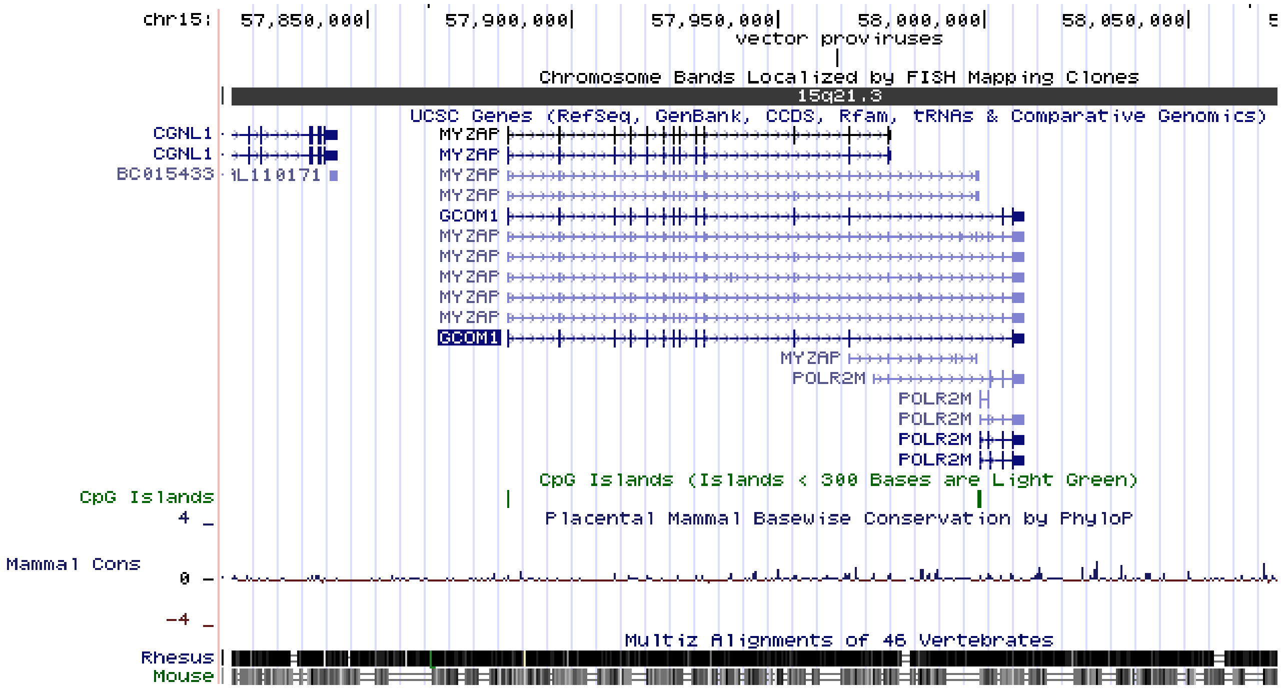

S4B. Proviral integration site in the GRINL1A complex locus 1 gene (*GCOM1*), also known as myocardial zonula adherens protein (*MYZAP*) on chromosome 15.

Supplemental Figure S4. Continued

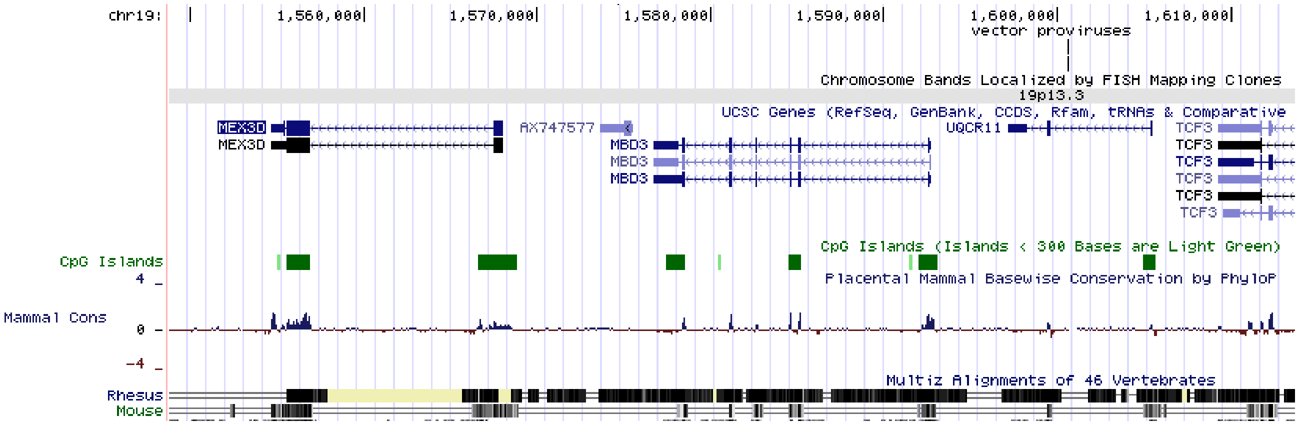

S4C. Proviral integration site near the mex-3 homolog D (*MEX3D*) on chromosome 19.

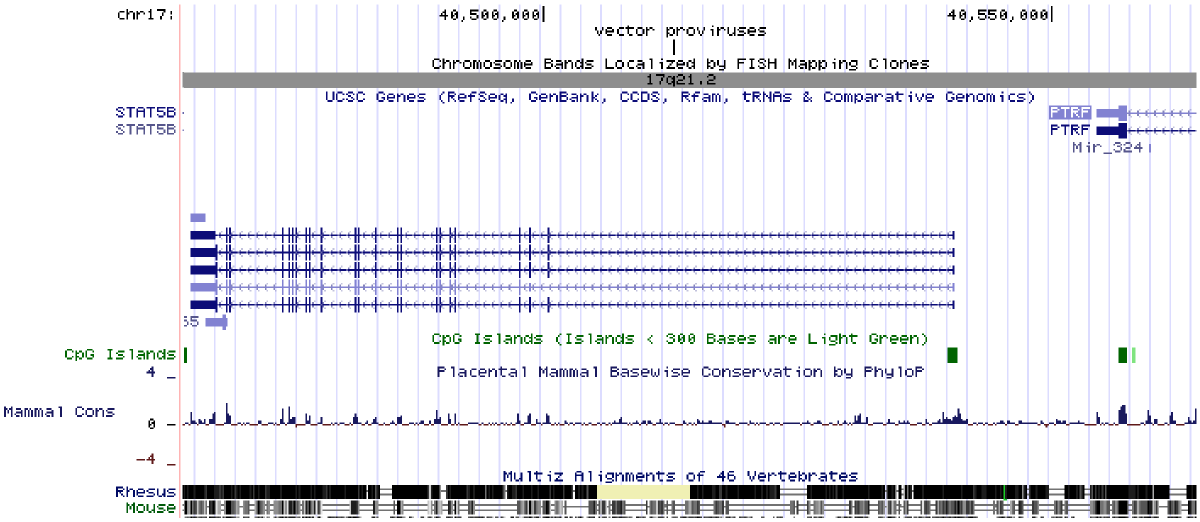

S4D. Proviral integration site near the polymerase 1 and transcript release factor gene (*PTRF*) on chromosome 17.

Supplemental Figure S4. Continued

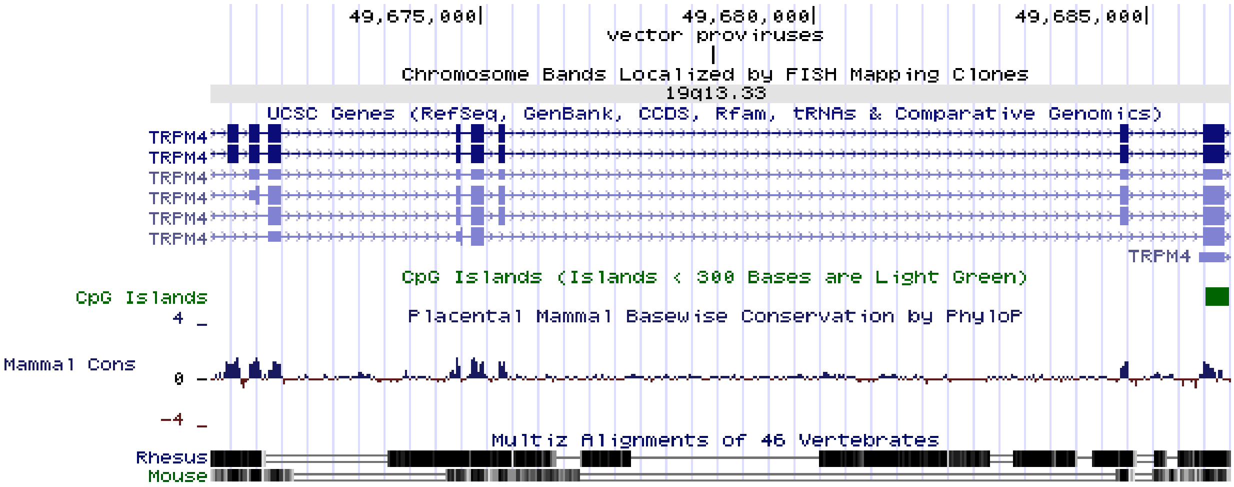

S4E. Proviral integration site in the transient receptor potential cation channel (*TRPM4*) on chromosome 1.
