# Supplementary material for: A novel approach to identify driver genes involved in androgen-independent prostate cancer
Source: Mol Cancer. 2014 May 23;13:120. doi: 10.1186/1476-4598-13-120 (PMC4098713; doi:10.1186/1476-4598-13-120)

Supplemental Figure S5. Screening of pGIPZ knockdown vectors for ATPAF1 and PTRF and confirmation of knockdown by RT-PCR

S5A. Screen for effective shRNAs

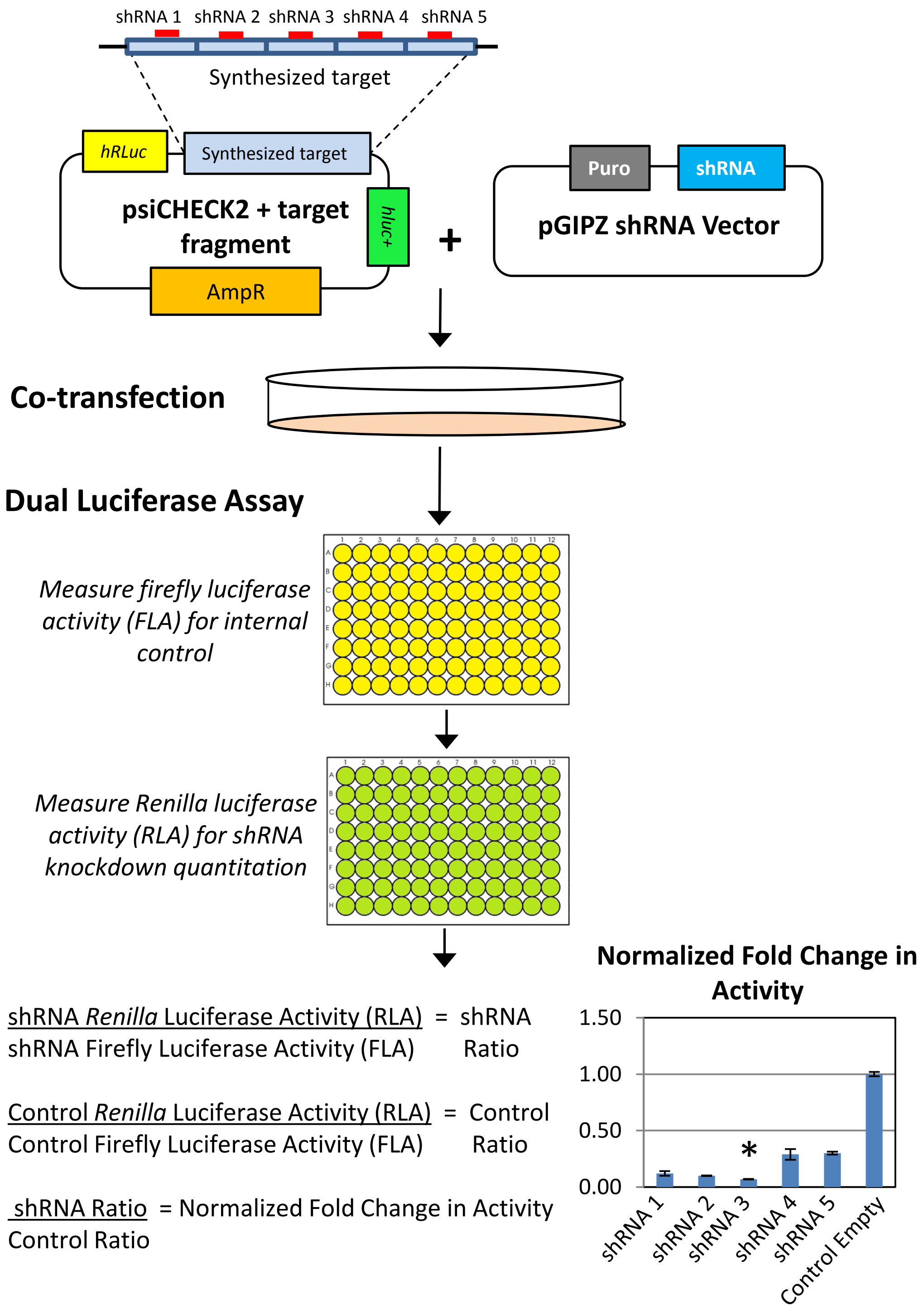

Supplemental Figure S5. Continued

S5B. Relative knockdown by each shRNA

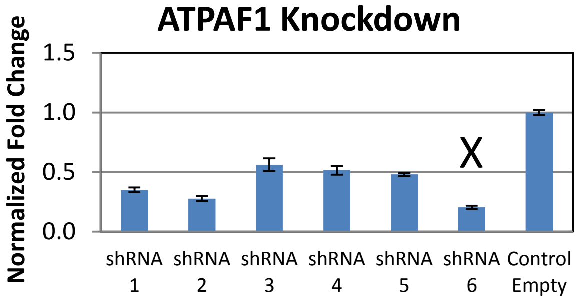

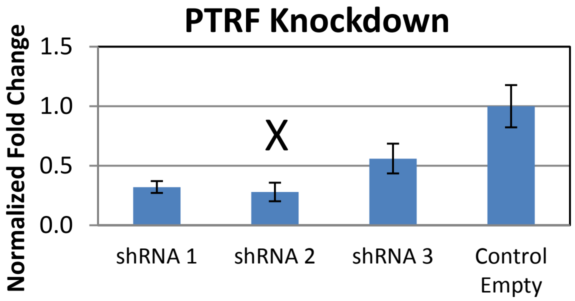

Gene	shRNA #	Clone ID	shRNA used:
ATPAF1	shRNA 1	V3LHS_344342	
	shRNA 2	V3LHS_344340	
	shRNA 3	V3LHS_402694	
	shRNA 4	V3LHS_344343	
	shRNA 5	V3LHS_344341	
	shRNA 6	V3LMM_507960	X
PTRF	shRNA 1	V3LHS_303433	
	shRNA 2	V3LHS_303432	X
	shRNA 3	V3LMM_502146	

S5C. Confirmation of knockdown by RT-PCR. LNCaP cells were transduced with lentiviruses carrying either empty vector (EV) or gene specific shRNA sequence (shATPAF1 or shPTRF). Total RNA isolated from the stably transduced LNCaP cells were subjected to reverse transcription -PCR analysis using gene specific primers, ATPAF1, PTRF and GAPDH. Top panel represents ATPAF1 level in LNCaP cells transduced with EV and shATPAF1. Bottom panel represents PTRF levels in LNCaP cells transduced with EV and shPTRF. GAPDH was used as loading control for normalization.

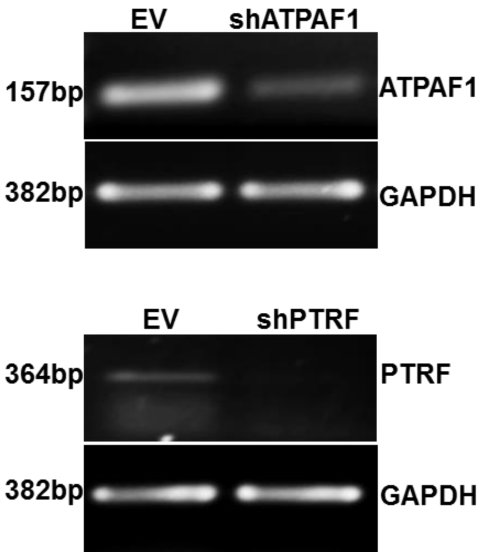

Supplement: Additional file 7: Figure S5 — Screening of pGIPZ knockdown vectors for ATPAF1 and PTRF and confirmation of knockdown by RT-PCR. [file 1476-4598-13-120-S7.pdf]
